# When chatbots are perceived as “understanding”: a study on how virtual agent persona styles and affective interaction promote mental health acceptance

**DOI:** 10.3389/fpsyg.2026.1863595

**Published:** 2026-09-03

**Authors:** Qi Wu, Shizhu Lu, Yuqi Yan, Lin Liu, Xiaojie Peng

**Affiliations:** 1 School of Art and Design, Guangdong University of Technology, Guangzhou, Guangdong, China; 2 Sichuan Fine Arts Institute, Chongqing, China; 3 Department of Design, Hanyang University, Seoul, Republic of Korea

**Keywords:** artificial intelligence, mental health chatbot, virtual agent persona, affective engagement, psychological acceptance, human–AI interaction

## Abstract

**Introduction:**

Mental health challenges among university students have become increasingly prevalent, while existing digital interventions often struggle to sustain user engagement and provide meaningful emotional support. Although previous studies have proposed various explanations for the limited acceptance of AI-based mental health chatbots, the role of affective experience remains insufficiently understood. This study proposes an affect-centered framework to examine how different virtual agent persona styles (Listener, Guide, and Motivator) influence university students’ psychological acceptance of mental health chatbots.

**Methods:**

Drawing on the Behavior Change Wheel (BCW) and the Unified Theory of Acceptance and Use of Technology (UTAUT), this study developed a dual-pathway model to investigate the mediating role of affective engagement in AI-based mental health chatbot acceptance. Data were collected from 266 university students who were randomly assigned to scenario-based chatbot stimuli representing three virtual agent persona styles. Partial least squares structural equation modeling (PLS-SEM), artificial neural network (ANN) analysis, and partial least squares multi-group analysis (PLS-MGA) were conducted to examine the proposed relationships and persona-related differences.

**Results:**

The results indicate that most proposed hypotheses were supported. Affective engagement emerged as a central mechanism linking persona-related characteristics and psychological acceptance. Multi-group analysis further revealed that although the overall acceptance mechanism remained largely consistent across the three persona conditions, significant differences existed in the strength of key relationships. Specifically, the effect of affective engagement on behavioral intention was stronger in the Guide and Motivator conditions than in the Listener condition.

**Discussion:**

These findings suggest that virtual agent persona styles do not fundamentally alter the psychological acceptance mechanism but can influence the strength of critical affective pathways. The study highlights the importance of affective engagement in AI-supported mental health interactions and provides practical implications for designing more adaptive, emotionally responsive, and user-centered mental health chatbots.

## 1 Introduction

In recent years, rapid advances in artificial intelligence and digital technologies have transformed the delivery of mental health services. This trend is particularly evident among university students, a population that experiences high and increasing levels of psychological distress (Hunt and Eisenberg, 2010; Blanco et al., 2008). According to the American College Health Association (2024), 76.4% of university students report moderate to high levels of stress, while 35.2% have sought mental health services within the past year. These statistics highlight a growing gap between mental health needs and available support resources, emphasizing the need for scalable and accessible intervention approaches (Erdur-Baker et al., 2006).

Digital mental health interventions have attracted increasing attention because of their accessibility, anonymity, potential scalability, and capacity to support greater user involvement (Bernaerts et al., 2024). Among these interventions, conversational AI chatbots have emerged as a promising approach for providing psychological support, particularly among digitally native populations such as university students (Balcombe, 2023; D’Alfonso et al., 2017). Despite their potential, the adoption and continued use of mental health chatbots remain limited. One possible explanation is that the emotional mechanisms underlying human–AI interaction are not yet fully understood.

Among the various factors influencing interaction outcomes, virtual agent persona style has been identified as a critical design element. Persona styles are constructed through combinations of visual appearance, linguistic characteristics, anthropomorphic cues, and social interaction strategies, all of which shape users’ emotional responses, trust perceptions, and interaction experiences (Abdelhalim et al., 2024; Abdulazeem and Hu, 2025; Chin and Yi, 2022; Gao et al., 2025; Go and Sundar, 2019; Li et al., 2025; Ruhland et al., 2015). Previous research suggests that socially supportive and empathetic agents can facilitate emotional disclosure and sustain engagement (Bickmore et al., 2005; Kang and Gratch, 2010; Lucas et al., 2014). However, most existing studies focus on isolated design features rather than examining how coherent persona styles influence users’ emotional experiences and subsequent acceptance of mental health chatbots. Furthermore, while anthropomorphic and empathic designs generally improve engagement, excessively realistic representations may evoke discomfort through the “uncanny valley” effect (Yuan et al., 2025). Such effects may be particularly relevant in mental health support contexts, where users often seek emotional reassurance and supportive interactions.

From a theoretical perspective, existing frameworks provide only partial explanations of user acceptance in mental health chatbot interactions. The Behavior Change Wheel (BCW) explains behavioral determinants through capability, opportunity, and motivation, yet provides limited insight into emotional experiences (Michie et al., 2014). Emotional Design Theory (EDT) explains how emotional responses emerge through visceral, behavioral, and reflective experiences but does not directly address technology acceptance (Norman, 2007). The Unified Theory of Acceptance and Use of Technology (UTAUT) focuses primarily on cognitive evaluations related to technology adoption and pays comparatively less attention to affective processes (Venkatesh et al., 2003). Consequently, the interplay between emotional experience and acceptance-related judgments remains insufficiently understood in mental health chatbot contexts. 

In the context of mental health chatbots, it is important to distinguish user experience from behavioral outcomes. User experience (UX) refers to users’ immediate perceptions and emotional responses during interaction, including affective engagement, perceived support, trust, and feelings of being understood. These experiential responses reflect how users interpret and emotionally evaluate their interaction with a chatbot. In contrast, behavioral intention refers to users’ willingness to continue using, recommend, or rely on the chatbot in the future. In the present study, behavioral intention is treated as the primary observable outcome representing users’ acceptance of mental health chatbots. Positive user experiences are expected to foster favorable acceptance-related evaluations and ultimately strengthen behavioral intention. Clarifying this distinction is important because many factors commonly examined in mental health chatbot research, such as engagement, trust, perceived support, and emotional resonance, belong conceptually to the domain of user experience rather than behavioral outcomes.

Several limitations remain in the existing literature. First, although persona design has received increasing attention, most studies focus on individual features such as anthropomorphism or empathy (Li et al., 2024; Meurs, 2021; Wang, 2025; Yuan et al., 2025), with relatively little attention given to systematic comparisons among different persona styles such as listener, guide, and motivator. Second, while emotional factors are widely recognized as important, the mechanisms through which persona styles influence behavioral intention through emotional arousal and affective engagement remain insufficiently explained. Third, the relationship between user experience and acceptance outcomes is often conceptually ambiguous, with constructs such as trust, support, engagement, and acceptance frequently used interchangeably. Fourth, existing studies typically rely on a single theoretical perspective, resulting in limited integration between emotional and cognitive explanations of acceptance.

To address these gaps, this study adopts an affect-oriented perspective to examine how different virtual agent persona styles influence users’ responses to mental health chatbots. Drawing upon EDT, BCW, and UTAUT, the study proposes an integrated framework in which emotional design factors and behavioral determinants influence affective engagement, which subsequently contributes to behavioral intention. Focusing on university students, three persona styles—listener, guide, and motivator—are compared to examine how different interaction styles shape emotional arousal, affective engagement, and acceptance-related outcomes. UTAUT and its subsequent extensions have been widely applied to explain behavioral intention and technology acceptance across consumer technology, mobile health, and AI-based health service contexts (Venkatesh et al., 2012; Yu et al., 2021; Su et al., 2025). In addition, the study investigates whether the proposed mechanism remains stable across persona conditions or varies in the strength of key psychological relationships. The study addresses the following research questions:

RQ1: How do virtual agent persona styles influence emotional arousal and affective engagement among university students?

RQ2: How does affective engagement contribute to behavioral intention toward mental health chatbots?

RQ3: How do emotional and cognitive factors jointly explain behavioral intention in mental health chatbot interactions?

This study aims to explain how persona styles shape users’ behavioral intention through affective engagement and acceptance-related evaluations. By integrating emotional and cognitive perspectives, the study contributes to a deeper understanding of affect-centered acceptance mechanisms and provides practical implications for the design of more adaptive and emotionally responsive mental health chatbots.

## 2 Literature review and hypothesis development

### 2.1 Virtual agent persona style and emotional design theory (EDT)

Virtual agents are digital entities that interact with users in human-like ways through speech, facial expressions, and bodily movements, and have been widely employed in contexts such as virtual reality and mental health applications (Pelachaud, 2009). In healthcare and psychological intervention settings, social presence generated through simulated social interactions has been shown to enhance user trust, technology acceptance, and perceived emotional support (Liebold and Ohler, 2013). Against this backdrop, the optimization of users’ affective experiences through design has emerged as a central research concern in virtual agent studies.

Among various design dimensions, stylization—defined as variations in appearance and behavior—has been recognized as a key mechanism that shapes users’ emotional responses (Canales et al., 2024). Empirical studies have shown that, compared with highly realistic agents, moderately stylized or non-realistic designs more effectively reduce perceived threat and evaluative anxiety, thereby enhancing users’ sense of safety and acceptance (Kukkakorpi and Pantti, 2021; Triberti et al., 2017). Specifically, Kukkakorpi and Pantti (2021) demonstrated that lower levels of realism can alleviate social evaluative pressure, while Triberti et al. (2017) found that non-realistic agents reduce perceived psychological threat, particularly among high-stress users.

However, existing research has largely conceptualized stylization as a single-dimensional design construct (e.g., visual realism or behavioral expression), with limited attention to a holistic perspective of persona construction. Recent studies have increasingly emphasized that visual and behavioral features do not operate in isolation but rather interact to form a coherent and consistent persona, thereby jointly shaping user experience (Abdelhalim et al., 2024; Ruhland et al., 2015). Building on this perspective, the present study extends stylization into a multidimensional construct referred to as persona style, defined as the integrated configuration of external appearance, interaction performance, and perceived social attributes. These elements are expected to work synergistically to influence users’ affective responses during human–AI interaction (Abdulazeem and Hu, 2025; Chin and Yi, 2022; Gao et al., 2025).

From the perspective of Emotional Design Theory (EDT), emotional responses are assumed to emerge across multiple levels, including visceral, behavioral, and reflective processing (Norman, 2013). Each level corresponds to a distinct type of design stimulus: external appearance primarily operates at the visceral level by shaping users’ initial emotional reactions through immediate perceptual cues; interaction performance functions at the behavioral level by influencing users’ ongoing experience and evaluation during interaction; and social perception is more closely associated with the reflective level, shaping users’ meaning-making processes and relational interpretations of the agent.

At the visceral level, prior research has suggested that semi-anthropomorphic or visually approachable designs can reduce psychological resistance and facilitate both emotional expression and sustained interaction (Do et al., 2024). Accordingly, when external design features convey warmth and approachability, positive affective responses are more likely to be evoked, thereby promoting deeper emotional engagement.

*H1*: Visceral-level design positively influences affective engagement.

At the behavioral level, interaction characteristics—including linguistic tone, response fluency, and consistency—are closely related to perceived interaction quality. Emotionally responsive and coherent interactions have been shown to increase trust and reduce uncertainty, thereby encouraging deeper user involvement (Amadou et al., 2023). Accordingly, higher levels of interaction quality are likely to enhance users’ emotional resonance and contribute to greater affective engagement.

*H2*: Behavioral-level design positively influences affective engagement.

At the reflective level, users’ perceptions of virtual agents represent a higher-level evaluative process through which interaction experiences are interpreted and assessed. Prior studies suggest that users’ reflective evaluations of virtual agents can influence emotional responses and sustained interaction (Bickmore et al., 2005; Kang and Gratch, 2010). Accordingly, positive reflective evaluations are expected to contribute to higher levels of affective engagement.

*H3*: Reflective-level design positively influences affective engagement.

### 2.2 Behavior change wheel theory(BCW)

The Behavior Change Wheel (BCW), developed by Susan Michie and colleagues, conceptualizes behavior as arising from interactions among capability, opportunity, and motivation. Its core component, the COM-B model, further conceptualizes behavior as resulting from the combined effects of capability, opportunity, and motivation (Michie et al., 2014). Through the integration of key elements drawn from multiple behavior change theories, the BCW has been extensively applied across a range of domains, including healthcare, education, and digital interventions (Tombor and Michie, 2017; West and Michie, 2020). The related Theoretical Domains Framework (TDF) further extends this framework by incorporating additional psychological and contextual factors, such as knowledge, skills, social context, and affective states, thereby expanding the explanatory scope of behavioral determinants (Michie et al., 2014; Shrestha et al., 2022).

Within digital mental health contexts, the BCW is widely used to examine the key enabling conditions and constraints that influence user participation in interventions. It has been shown to be particularly valuable in explaining why individuals may fail to maintain sustained engagement in mental health–related activities (Fancourt et al., 2020). Specifically, Fancourt et al. (2020) found that limitations in capability (e.g., lack of coping skills), restricted opportunities (e.g., insufficient social support), and reduced motivation (e.g., depressive affect) can significantly reduce the likelihood of engagement in mental health activities.

To better account for affective processes in human–AI interaction contexts, some studies have reconceptualized the notion of “automatic motivation” within the BCW by contextualizing it as emotional arousal. Emotional arousal can be understood as an immediate state of psychological activation triggered by interface design features or anthropomorphic cues (Rahaman et al., 2023). This conceptual refinement extends the applicability of the BCW to interactive contexts and is theoretically aligned with the visceral-level emotional responses described in Emotional Design Theory proposed by Norman (2007).

Empirical evidence provides further support for this mechanism. For instance, Félix et al. (2019) demonstrated in experimental settings that interfaces capable of expressing affective cues can significantly enhance user trust and interaction quality, primarily through the amplification of users’ emotional responses. Similarly, studies by Calvo and D’Mello (2010) and Shen et al. (2009) suggest that emotion-oriented feedback mechanisms promote deeper cognitive and affective involvement. Collectively, these findings suggest that emotional arousal serves as a critical mediating mechanism between design features and user engagement, thereby highlighting the relevance of the BCW in explaining the development of affective engagement in interaction contexts. This conceptualization should be regarded as an interpretive adaptation of the BCW framework rather than a direct representation of the original construct proposed by Michie et al. (2014).

In the present study, the BCW is adopted as an interpretive framework rather than as a direct predictive model. Capability and opportunity are treated as enabling conditions for user engagement, whereas automatic motivation is operationalized in terms of emotional arousal. Based on this framework, the following hypotheses are proposed:

*H4*: Behavioral capability positively influences affective engagement (AE).

*H5*: Perceived opportunity positively influences affective engagement. (AE).

*H6*: Emotional arousal positively influences affective engagement. (AE).

### 2.3 Unified theory of acceptance and use of technology (UTAUT)

The Unified Theory of Acceptance and Use of Technology (UTAUT) proposes that users’ intentions to adopt and use a technology are primarily determined by performance expectancy, effort expectancy, social influence, and facilitating conditions (Venkatesh et al., 2003). UTAUT has been widely applied to explain technology acceptance across various digital health and human–computer interaction contexts.

In the context of mental health chatbot interactions, it is important to distinguish affective engagement from behavioral intention. Affective engagement refers to users’ emotional involvement, immersion, and experiential connection during interaction with the chatbot and is therefore conceptualized as a user-experience construct. Behavioral intention, in contrast, represents users’ willingness to continue using, recommend, or rely on the chatbot in the future. In the present study, behavioral intention serves as the operational representation of users’ psychological acceptance toward mental health chatbots.

Accordingly, affective engagement is conceptualized as an experiential process variable, whereas behavioral intention represents the primary acceptance-related outcome variable. Although affective engagement may contribute to behavioral intention, the two constructs capture different stages of users’ responses to chatbot interactions.

Performance expectancy refers to the degree to which users believe that a chatbot can effectively support their psychological needs and improve their well-being. Prior studies have consistently shown that perceived usefulness positively influences technology adoption intentions (Venkatesh et al., 2003). Therefore:

*H7*: Performance expectancy positively influences behavioral intention toward mental health chatbots.

Effort expectancy refers to the perceived ease of using and interacting with a chatbot system. Technologies that are easier to understand and operate are generally associated with higher acceptance intentions (Davis, 1989; Venkatesh et al., 2003). Therefore:

*H8*: Effort expectancy positively influences behavioral intention toward mental health chatbots.

Social influence reflects the extent to which individuals perceive that important others encourage or support the use of a technology. In mental health contexts, recommendations from peers, family members, or institutions may positively shape users’ willingness to adopt chatbot-based services. Therefore:

*H9*: Social influence positively influences behavioral intention toward mental health chatbots.

Facilitating conditions refer to users’ perceptions of the availability of resources, technical support, and environmental conditions that enable technology use. Prior research suggests that supportive conditions can increase users’ confidence and willingness to adopt digital systems. Therefore:

*H10*: Facilitating conditions positively influence behavioral intention toward mental health chatbots.

### 2.4 The role of affective engagement in mental health chatbot acceptance

Affective engagement reflects users’ emotional involvement, attentional investment, and experiential connection during interaction with a chatbot. Within emotionally sensitive contexts such as mental health support, affective engagement may play a particularly important role because users often evaluate digital support systems not only in terms of utility but also in terms of emotional resonance and interaction quality.

Higher levels of affective engagement are often associated with perceptions that interactions are more natural, understandable, and easy to follow. In mental health chatbot contexts, users who feel emotionally engaged may perceive the interaction process as smoother and less effortful. Accordingly, affective engagement may be related to users’ perceptions of effort expectancy.

Although effort expectancy is traditionally treated as an antecedent of technology acceptance, recent human–AI interaction research suggests that affective responses and usability perceptions may be closely interconnected. Therefore, the present study specifies the relationship between affective engagement and effort expectancy as a theoretically informed association within the proposed model. However, given the cross-sectional nature of the study, this specification should not be interpreted as definitive evidence of causal direction.

*H11*: Affective engagement is positively associated with effort expectancy toward mental health chatbots.

In addition, affective engagement may directly contribute to users’ acceptance-related responses. Users who feel emotionally connected to and engaged with a chatbot are more likely to develop favorable evaluations and stronger intentions to continue using the system. Therefore:

*H12*: Affective engagement positively influences behavioral intention toward mental health chatbots.

### 2.5 Hypothetical model

**Figure 1:**
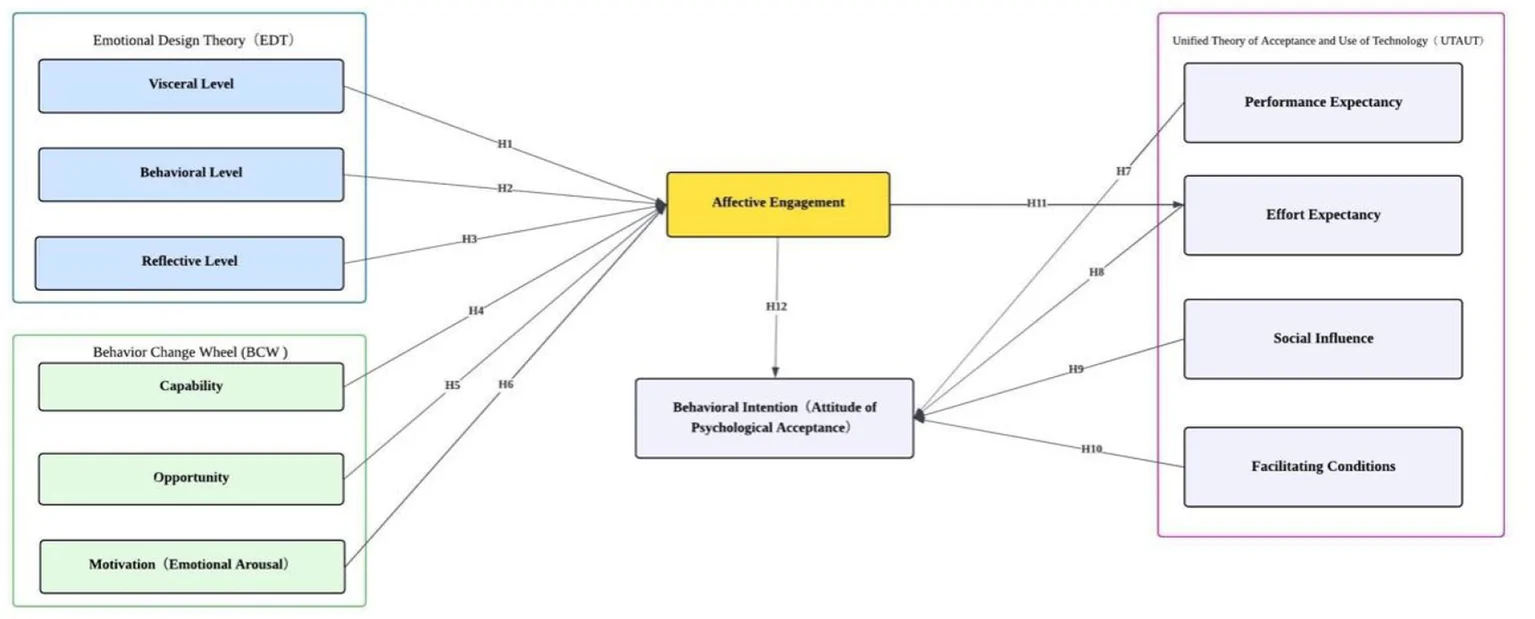
Hypothesized path model. Behavioral Intention (BI) serves as the operational representation of users’ psychological acceptance toward mental health chatbots. Within the BCW framework, Motivation (MO) is conceptually adapted as Emotional Arousal, which facilitates Affective Engagement (AE).

This study integrates Emotional Design Theory (EDT), the Behavior Change Wheel (BCW), and the Unified Theory of Acceptance and Use of Technology (UTAUT) to propose a model linking persona style, emotional arousal, affective engagement, and behavioral intention (Figure 1). EDT explains emotional responses across different design levels, BCW captures the antecedent conditions of engagement, and UTAUT explains acceptance-related outcomes. Within the proposed framework, emotional arousal and affective engagement are regarded as key mechanisms influencing behavioral intention toward mental health chatbots.

## 3 Research methodology

### 3.1 Research procedure and selection of experimental tools and platforms

**Figure 2:**
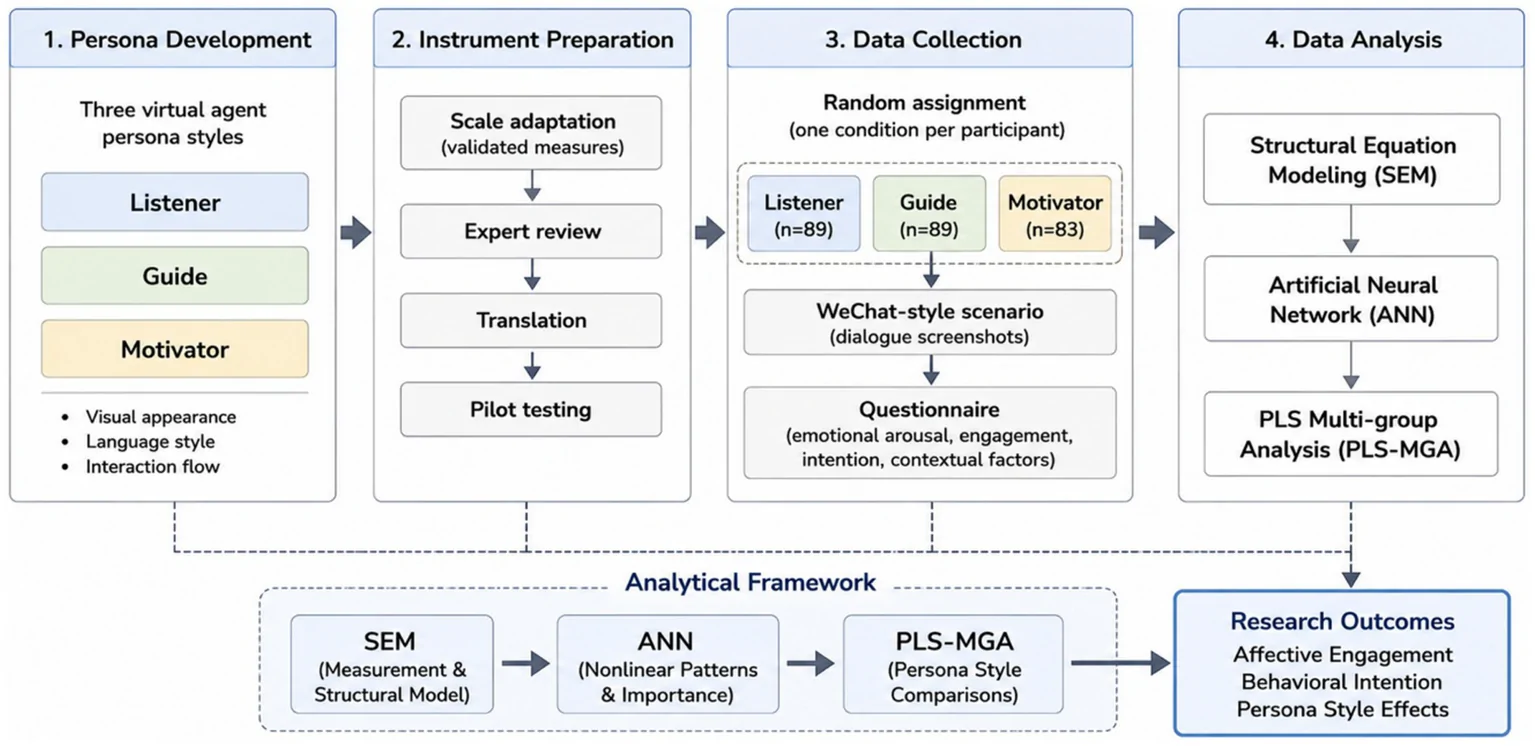
Research procedure and analytical framework.

This study adopted a scenario-based experimental survey design to examine how university students responded emotionally and cognitively to different virtual agent persona styles in a mental health context. As outlined in Figure 2, the research process consisted of four stages: persona development, instrument preparation, data collection, and data analysis.

Three types of virtual agent personas—Listener, Guide, and Motivator—were developed to represent distinct interaction orientations rather than stable personality traits (Liew et al., 2023; Mori et al., 2012). The differences between these conditions were operationalized through coordinated variations in visual appearance, language style, and interaction flow, allowing each persona to be clearly distinguished within the experimental scenarios.

The study was implemented using the Wenjuanxing survey platform and WeChat-style dialogue interfaces. Participants were randomly assigned to one of three persona conditions, with each participant being exposed to only a single condition to avoid carryover effects. Rather than engaging with a live chatbot, participants were presented with standardized persona-based dialogue scenarios in the form of WeChat-style chat screenshots.

After reviewing the assigned scenario materials, participants completed a questionnaire assessing emotional arousal, affective engagement, behavioral intention, and related contextual factors. The measurement items were derived from validated scales and adapted to the mental health context. The questionnaire was further refined through expert review, translation procedures, and pilot testing prior to the main study.

The analytical procedure consisted of three stages. First, structural equation modeling (SEM) was used to evaluate the measurement model and test the proposed structural relationships. Second, artificial neural network (ANN) analysis was conducted on significant predictors identified in the SEM results to capture nonlinear patterns and assess their relative contribution to behavioral intention. Third, partial least squares multi-group analysis (PLS-MGA) was performed to examine whether the proposed relationships differed significantly across the three persona conditions (Listener, Guide, and Motivator). Given that participants were randomly assigned to one of the three persona groups and that persona style represented the central experimental manipulation of this study, PLS-MGA was employed to compare path coefficients across groups and determine whether the strength of the proposed relationships varied according to persona style. This additional analysis provided direct evidence regarding the comparative effects of different persona styles on the proposed acceptance mechanism.

Overall, the study design aimed to maintain a balance between experimental control and ecological validity, thereby providing a rigorous basis for examining how persona styles influence users’ emotional responses and behavioral intentions in mental health chatbot scenarios.

#### 3.1.1 Development of experimental stimuli

To ensure experimental control while maintaining ecological validity, this study employed a scenario-based experimental design rather than a real-time chatbot system. The experimental stimuli were developed through a multi-stage process. First, initial dialogue drafts were generated using GPT-assisted prompts within a mental health support context. These drafts were subsequently reviewed, refined, and standardized by the research team according to the theoretical characteristics of the three target persona styles: Listener, Guide, and Motivator (Table 1).

**Table 1:** Design characteristics of the three persona conditions.

Persona style	Role	Communication	Strategy	Example response
Listener	Emotional companion	Warm, empathetic, and supportive	Emotional validation and active listening	“I understand how overwhelming this situation may feel for you. It is completely understandable to feel stressed.”
Guide	Problem-solving assistant	Structured, analytical, and goal-oriented	Cognitive guidance and practical suggestions	“Let us break this issue into smaller steps and identify what can be addressed first.”
Motivator	Encouraging coach	Positive, energetic, and action-oriented	Encouragement and motivational reinforcement	“You have already taken an important step by seeking support. I believe you can handle this challenge.

Across all conditions, the dialogue topic, conversational structure, information content, and number of interaction rounds were kept constant. Only persona-specific communication styles and feedback strategies were manipulated.

Across all conditions, the dialogue topic, conversational structure, informational content, and number of dialogue rounds were held constant. Only persona-specific communication styles and feedback strategies were manipulated. Specifically, the Listener emphasized empathy, emotional validation, and active listening; the Guide focused on structured guidance and problem-solving support; and the Motivator adopted an encouraging and action-oriented communication style.

The study did not employ a live generative chatbot during data collection. Instead, GPT was used solely during the stimulus development stage to assist in generating initial dialogue drafts, which were subsequently standardized and fixed prior to participant exposure. Thus, all participants were exposed to identical scenario materials within each experimental condition.

Each persona condition consisted of a standardized six-round dialogue scenario. Following final revision, the dialogues were converted into WeChat-style chat screenshots to enhance realism and facilitate participant immersion in the scenario. The screenshots were embedded into the Wenjuanxing online survey platform and presented as experimental stimuli. Participants were instructed to carefully review the assigned dialogue scenario before completing the questionnaire.

This design ensured consistency of stimulus exposure across participants while enabling systematic manipulation of persona style. A summary of the dialogue structure is presented in Table 2, and the complete dialogue scripts for all three persona conditions are provided in Supplementary File S1 to facilitate transparency and replication.

**Table 2:** Structure of the standardized six-round dialogue.

Dialogue round	Interaction purpose	Example content
Round 1	Greeting and rapport building	Initial welcome and emotional check-in
Round 2	Problem disclosure	Student describes stress-related concerns
Round 3	Emotional response	Persona-specific reaction to the concern
Round 4	Reflection and support	Emotional validation, guidance, or encouragement
Round 5	Coping strategy	Suggested coping actions or perspectives
Round 6	Closing interaction	Summary and supportive closing message

#### 3.1.2 Experimental procedure

**Figure 3:**
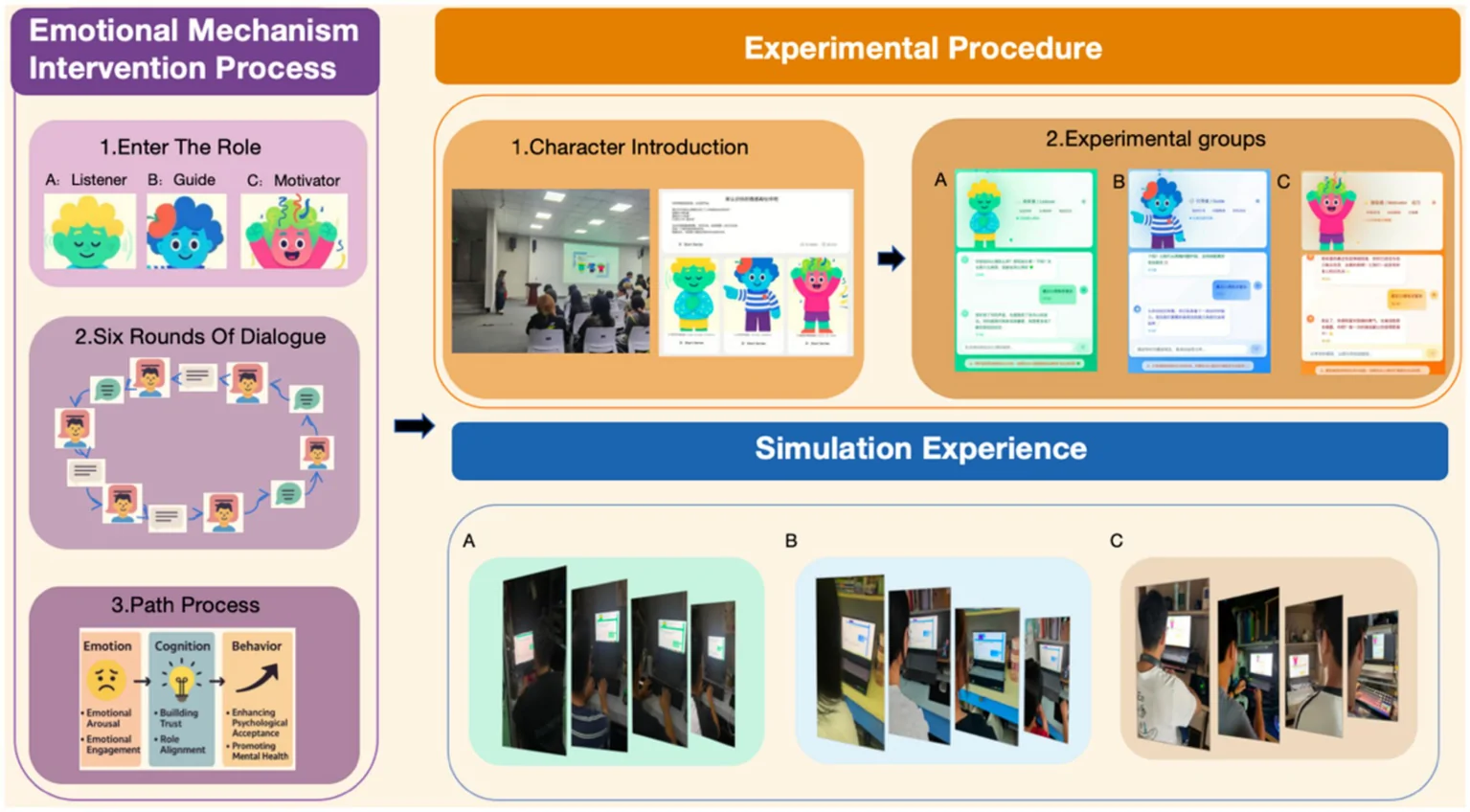
Development and presentation of persona-based experimental stimuli.

This study employed a scenario-based between-subjects experimental design to examine the effects of three virtual agent persona styles (Listener, Guide, and Motivator), as illustrated in Figure 3. Participants first provided informed consent and were then randomly assigned through the Wenjuanxing platform to one of the three persona conditions. Each participant reviewed only one WeChat-style dialogue scenario to avoid carryover effects.

The persona manipulation was implemented through differences in visual presentation, communication style, and interaction strategy. The Listener emphasized empathy and emotional support, the Guide focused on structured guidance and problem-solving, and the Motivator adopted an encouraging and action-oriented style. To control for potential confounding effects, the dialogue topic, structure, content, and length were kept constant across conditions, while only persona-related expressions and feedback strategies varied.

All materials were reviewed by experts in mental health and human–computer interaction. Prior to the main study, a pilot test was conducted with 18 university students, who were randomly assigned to the Listener (*n* = 6), Guide (*n* = 6), or Motivator (*n* = 6) condition. After reviewing the dialogue materials, participants were asked to identify the role portrayed by the chatbot and provide feedback regarding the clarity of the persona characteristics. The results indicated that the three persona styles were generally distinguishable. Minor revisions were subsequently made to the wording and persona-specific expressions before the materials were finalized.

Following exposure to the assigned scenario, participants completed a questionnaire measuring emotional arousal, affective engagement, effort expectancy, behavioral intention, and related contextual variables. A standardized procedure was adopted across all conditions to ensure experimental consistency while maintaining ecological validity.

### 3.2 Development of the questionnaire and recruitment of participants

#### 3.2.1 Development of the questionnaire

The questionnaire used in this study was designed to assess university students’ emotional responses, their levels of affective engagement, and psychological acceptance attitudes during interactions with AI chatbots of different persona styles. Multiple item formats, including semantic differential scales and Likert-type scales, were used to capture participants’ subjective experiences throughout the interaction process.

The measurement items were not derived from a single theoretical source but were constructed and adapted from prior studies on virtual agents, affective interaction, and technology acceptance. Constructs related to emotional arousal and engagement were informed by research in affective computing and human–computer interaction, whereas measures such as psychological acceptance, performance expectancy, effort expectancy, social influence, and facilitating conditions were derived from established technology acceptance frameworks and modified for the mental health setting.

In adapting these items, emphasis was placed on contextual relevance and content validity rather than strict replication. The instrument was reviewed by experts in mental health and human–computer interaction and subsequently refined through translation procedures and pilot testing to ensure clarity and suitability for university students.

The final questionnaire included measures of perceived virtual agent characteristics, emotional arousal, affective engagement, behavioral intention, and relevant contextual variables, thus providing a robust measurement foundation for subsequent structural equation modeling (SEM), artificial neural network (ANN), and partial least squares multi-group analysis (PLS-MGA) (Table 3).

**Table 3:** Measurement variables and their references.

Dimension	Code and content	Source
Visceral level (VL)	VL1: The chatbot gives an overall impression of warmth.	Choi et al. (2024), Liew et al. (2023), and Norman (2013)
	VL2: The chatbot demonstrates a tendency to understand my emotions.	
	VL3: The chatbot guides the conversation in a purposeful manner.	
	VL4: The chatbot conveys a sense of equality during the conversation.	
	VL5: The chatbot proactively expresses its own standpoint.	
	VL6: The chatbot conveys an intention to provide emotional support.	
Behavioral level (BL)	BL1: The interface of this chatbot is highly intuitive to use.	Liew et al. (2023), Luger and Sellen (2016), and McLean and Osei-Frimpong (2019)
	BL2: The interaction process with the chatbot is smooth and seamless.	
	BL3: I feel that operating this chatbot is effortless and free of burden.	
	BL4: The chatbot responds promptly to my inputs, making the interaction process satisfactory.	
Reflective level of affective engagement (RL)	RL1: I intend to continue using this chatbot in the future.	Norman (2013) and Park et al. (2023)
	RL2: I will frequently use it when I have emotional needs.	
	RL3: I would recommend it to others.	
	RL4: It will become part of my psychological self-help resources.	
Capability(CP)	CP: I know how to manage my emotions or stress through this chatbot.	Timmermans et al. (2024)
	CP: I possess the basic capability to regulate my emotions using the chatbot.	
	CP: Conversing with this chatbot makes me more aware of my emotional state.	
Opportunity (OP)	OP: My daily environment allows me to conveniently use this chatbot.	Algumaei et al. (2025) and Timmermans et al. (2024)
	OP: I have access to the devices and internet connectivity required to use this chatbot.	
	OP: My friends or colleagues also use, or support the use of, similar chatbots.	
Motivation (MO)	MO1: During the interaction, I felt my emotions being stimulated.	(Rahaman et al., 2023; Sun et al., 2024)
	MO2: The chatbot elicited a strong emotional response from me.	
	MO3: I experienced heightened emotional arousal.	
	MO4: This interaction made me feel energized.	
Behavioral intention (BI)	BI1: I intend to continue using this chatbot in the future.	Park et al. (2023) and Venkatesh et al. (2003)
	BI2: I will frequently use this chatbot when I have emotional needs.	
	BI3: I will recommend this chatbot to others.	
	BI4: This chatbot will become part of my psychological self-help routine.	
Effort expectancy (EE)	EE1: Learning to use this chatbot is easy for me.	Venkatesh et al. (2003)
	EE2: I find this chatbot easy to use.	
	EE3: The interaction process is highly intuitive.	
Performance expectancy (PE)	PE1: Using this chatbot helps improve my emotional state.	Sabour et al. (2023) and Venkatesh et al. (2003)
	PE2: It assists me in managing stress or negative emotions.	
	PE3: This chatbot is useful for supporting mental health.	
Social influence (SI)	SI1: People who are important to me think I should use this chatbot.	Cao et al. (2022) and Yamaguchi et al. (2019)
	SI2: People around me would support my use of this chatbot.	
Facilitating conditions (FC)	FC1: I have the necessary resources to use this chatbot	Venkatesh et al. (2003)
	FC2: This chatbot is well-adapted to the devices and tools I commonly use.	
Affective engagement (AE)	AE1: I feel emotionally engaged during interactions with the chatbot.	Liew et al. (2023) and Purington et al. (2017)
	AE2: I am highly focused when conversing with the chatbot.	
	AE3: I feel a certain sense of connection with the chatbot during interactions.	
	AE4: The experience with this chatbot gives me a strong sense of emotional involvement.	
	AE5: Interacting with this chatbot creates an emotional attachment for me.	

Reflective-level items are modeled as a reflective subdimension of affective engagement, capturing users’ post-interaction evaluative appraisal rather than behavioral intention.

#### 3.2.2 Recruitment of participants

Participants were recruited through an online student forum at a comprehensive university, yielding 336 responses. Using a between-subjects design, participants were randomly assigned via the Wenjuanxing platform to one of three persona conditions: Listener, Guide, or Motivator. The platform’s built-in randomization function ensured a balanced allocation across groups.

After data screening, 266 valid responses were retained for analysis, including 88 participants in the Listener condition, 89 in the Guide condition, and 89 in the Motivator condition. The sample was relatively balanced in gender (54.1% female) and consisted primarily of undergraduate students aged 19–21 years (60.9%). Overall, the sample was consistent with the target population of university students (see Table 4).

**Table 4:** Demographic characteristics of respondents.

**Attribute**	**Items**	**Numbers**	**Percentage (%)**
Gender	Male	122	45.9
	Female	144	54.1
Age	≤18 years	18	6.8
	19–21 years	162	60.9
	22–24 years	56	21.1
	25–27 years	21	7.9
	≥28 years	9	3.4
Academic status	Undergraduate, Year 1	36	13.5
	Undergraduate, Year 2	81	30.5
	Undergraduate, Year 3	66	24.8
	Undergraduate, Year 4	31	11.7
	Graduate (Master’s/Other)	52	19.5
Field of study	Science and Engineering	114	42.9
	Humanities	67	25.2
	Arts and Design	44	16.5
	Medical Sciences	28	10.5
	Other	13	4.9
Use of mental health apps	Never	82	30.8
	Occasionally	126	47.4
	Frequently	58	21.8
Perceived stress in the past 30 days	Minimal stress	38	14.3
	Mild stress	73	27.4
	Moderate stress	84	31.6
	Considerable stress	44	16.5
	Considerable stress	27	10.2

## 4 Data analysis

### 4.1 Measurement model assessment

The quality of the measurement model was assessed through multiple criteria, including collinearity, reliability, convergent validity, and discriminant validity (see Tables 5, 6). Because all constructs were measured using a single self-report questionnaire administered immediately after exposure to the experimental stimuli, additional attention was given to the potential influence of common method bias. Following Kock (2015), a full-collinearity assessment was conducted by examining variance inflation factor (VIF) values for all latent constructs. The VIF values ranged from 1.000 to 2.896, remaining below the recommended threshold of 3.3. These results suggest that neither multicollinearity nor substantial common method bias was likely to threaten the validity of the findings. Nevertheless, as all variables were collected from the same respondents at a single point in time, method-related variance cannot be completely ruled out.

**Table 5:** Path coefficients, significance levels, and VIF values.

Reflective Components	Items	Factor Loading	Cronbach’s alpha (α)	CR	AVE
AE	AE 1	0.874	0.909	0.914	0.734
	AE 2	0.876			
	AE 3	0.844			
	AE 4	0.904			
	AE 5	0.779			
BI	BI 1	0.903	0.874	0.877	0.726
	BI 2	0.866			
	BI 3	0.811			
	BI 4	0.825			
BL	BL 1	0.836	0.871	0.873	0.722
	BL 2	0.879			
	BL 3	0.837			
	BL 4	0.845			
CP	CP 1	0.915	0.903	0.906	0.837
	CP 2	0.915			
	CP 3	0.915			
EE	EE 1	0.915	0.865	0.869	0.788
	EE 2	0.887			
	EE 3	0.860			
FC	FC 1	0.933	0.922	0.922	0.856
	FC 2	0.917			
MO	MO 1	0.894	0.900	0.904	0.769
	MO 2	0.872			
	MO 3	0.876			
	MO 4	0.867			
OP	OP 1	0.903	0.804	0.820	0.722
	OP 2	0.888			
	OP 3	0.749			
PE	PE 1	0.917	0.905	0.906	0.840
	PE 2	0.917			
	PE 3	0.915			
RL	RL 1	0.914	0.929	0.930	0.825
	RL 2	0.902			
	RL 3	0.896			
	RL 4	0.922			
SI	SI 1	0.926	0.842	0.927	0.864
	SI 2	0.932			
VL	VL 1	0.802	0.886	0.889	0.637
	VL 2	0.809			
	VL 3	0.778			
	VL 4	0.826			
	VL 5	0.749			
	VL 6	0.822			

AE = Affective Engagement; BI = Behavioral Intention; BL = Behavioral-Level Design; CP = Capability; EE = Effort Expectancy; FC = Facilitating Conditions; MO = Emotional Arousal; OP = Opportunity; PE = Performance Expectancy; RL = Reflective-Level Design; SI = Social Influence; VL = Visceral-Level Design.

**Table 6:** Inter-structural correlation matrix, HTMT statistic.

Constructs	AE	BI	BL	CP	EE	FC	MO	OP	PE	RL	SI	VL
AE	**0.857**	0.756	0.703	0.667	0.691	0.457	0.748	0.594	0.702	0.727	0.606	0.738
BI	0.844	**0.852**	0.612	0.613	0.728	0.580	0.739	0.526	0.732	0.721	0.708	0.673
BL	0.784	0.697	**0.850**	0.524	0.683	0.407	0.655	0.595	0.608	0.637	0.458	0.606
CP	0.731	0.687	0.587	**0.915**	0.618	0.400	0.597	0.390	0.593	0.622	0.483	0.622
EE	0.776	0.835	0.784	0.698	**0.888**	0.435	0.676	0.537	0.676	0.671	0.521	0.644
FC	0.524	0.678	0.477	0.461	0.513	**0.925**	0.496	0.474	0.527	0.526	0.543	0.420
MO	0.820	0.829	0.734	0.656	0.761	0.571	**0.877**	0.602	0.641	0.741	0.691	0.658
OP	0.690	0.629	0.705	0.460	0.636	0.583	0.707	**0.849**	0.469	0.519	0.450	0.443
PE	0.769	0.820	0.683	0.655	0.762	0.608	0.706	0.551	**0.917**	0.701	0.613	0.697
RL	0.784	0.797	0.706	0.677	0.749	0.599	0.808	0.604	0.764	**0.908**	0.624	0.760
SI	0.692	0.824	0.530	0.551	0.609	0.648	0.792	0.561	0.702	0.705	**0.929**	0.574
VL	0.815	0.762	0.685	0.690	0.731	0.491	0.730	0.522	0.775	0.837	0.664	**0.798**

Diagonal values (in bold) represent $\sqrt{\mathrm{AVE}}$ , values above the diagonal represent inter-structural correlations, and values below the diagonal represent the HTMT statistics.

The indicators demonstrated satisfactory reliability. All factor loadings exceeded the recommended threshold of 0.70, indicating acceptable indicator reliability. Measures of internal consistency were also satisfactory, with both Cronbach’s *α* and composite reliability (CR) values exceeding the recommended cutoff of 0.70 (Hair et al., 2021). Convergent validity was further supported, as all constructs exhibited average variance extracted (AVE) values above 0.50, satisfying the recommended criteria proposed by Fornell and Larcker (1981). The detailed results are presented in Table 5.

Discriminant validity was evaluated using both the heterotrait–monotrait ratio (HTMT) and the Fornell–Larcker criterion. The results indicate that all HTMT values were below the recommended threshold of 0.85 (Henseler et al., 2015). In addition, the square root of the AVE for each construct exceeded its correlations with all other constructs, providing further evidence of adequate discriminant validity (Fornell and Larcker, 1981). These findings collectively support the reliability and validity of the measurement model, with detailed statistics reported in Table 6.

Although affective engagement and behavioral intention are theoretically related, they represent conceptually distinct constructs in the present study. Affective engagement captures users’ emotional involvement and experiential connection during chatbot interaction, whereas behavioral intention reflects users’ future intentions to continue using, recommend, or rely on the chatbot. The satisfactory HTMT and Fornell–Larcker results provide empirical evidence that affective engagement and behavioral intention are empirically distinguishable despite their theoretical relatedness.

Discriminant validity was evaluated using both the HTMT ratio and the Fornell–Larcker criterion. The results indicate that all HTMT values did not exceed the recommended threshold of 0.85 (Henseler et al., 2014). In addition, the square root of the average variance extracted (AVE) for each construct was greater than its correlations with other constructs (Fornell and Larcker, 1981). These findings further confirm adequate discriminant validity among the latent constructs, with detailed statistics reported in Table 6.

The explanatory and predictive capabilities of the structural model were further examined. With respect to explanatory power, the *R*^2^ value for AE was 0.734 and for BI was 0.750, both indicating substantial explanatory strength, whereas the *R*^2^ value for EE was 0.479, suggesting a moderate level (Chin, 1998). Predictive relevance was assessed using the Stone–Geisser *Q*^2^ statistic. The *Q*^2^ values were 0.702 for AE, 0.720 for BI, and 0.562 for EE. All values exceeded zero and fell within a moderate-to-high range, indicating that the model demonstrates satisfactory predictive capability (Sarstedt et al., 2021). The *R*^2^ and *Q*^2^ values of the endogenous variables are presented in Table 7.

**Table 7:** *R*^2^ and *Q*^2^ values of endogenous constructs.

Constructs	*R* ^2^	Remarks	*Q* ^2^	Predictive relevance
AE	0.734	strong	0.702	Strong
BI	0.750	strong	0.720	Strong
EE	0.479	moderate	0.562	Strong

Effect sizes (ƒ^2^) were used to assess the relative contribution of each construct to the explained variance of endogenous variables (Henseler et al., 2014). Following Cohen’s (2013) guidelines, ƒ^2^ values of 0.02–0.15, 0.15–0.35, and above 0.35 indicate small, medium, and large effects, respectively. The results showed that affective engagement exerted a large effect on effort expectancy (AE → EE, ƒ^2^ = 0.918). In contrast, the significant paths SI → BI (ƒ^2^ = 0.130), EE → BI (ƒ^2^ = 0.120), AE → BI (ƒ^2^ = 0.102), VL → AE (ƒ^2^ = 0.090), CP → AE (ƒ^2^ = 0.070), BL → AE (ƒ^2^ = 0.060), and MO → AE (ƒ^2^ = 0.060) demonstrated small effect sizes. The effects of FC → BI (ƒ^2^ = 0.045), PE → BI (ƒ^2^ = 0.036), and OP → AE (ƒ^2^ = 0.036) were also small, indicating relatively limited contributions to the explained variance of the endogenous constructs. The path RL → AE was not statistically significant and therefore did not exhibit a meaningful effect size.

Overall, the findings suggest that the explanatory power of behavioral intention (BI) was primarily associated with effort expectancy, social influence, and affective engagement, whereas affective engagement (AE) was mainly influenced by visceral-level design, capability, behavioral-level design, and emotional arousal. Model fit was evaluated using the standardized root mean square residual (SRMR), which yielded a value of 0.067, indicating an acceptable level of model fit (Hu and Bentler, 1999). The GoF index was not reported due to its limited suitability for evaluating PLS-SEM models (Henseler and Sarstedt, 2013). Mediation analysis further revealed that affective engagement significantly mediated the relationship between emotional arousal and behavioral intention (*β* = 0.055, *p* < 0.05), suggesting that emotional arousal influences behavioral intention indirectly through affective engagement (Table 8).

**Table 8:** Mediation analysis of MO → AE → BI.

Indirect effect path	Original sample (β)	Standard deviation	*T* statistics	*p-*values
MO → AE → BI	0.055	0.025	2.218	*

***p < 0.001, **p < 0 0.01, *p < 0.05.

### 4.2 Model fit and hypothesis testing

**Figure 4:**
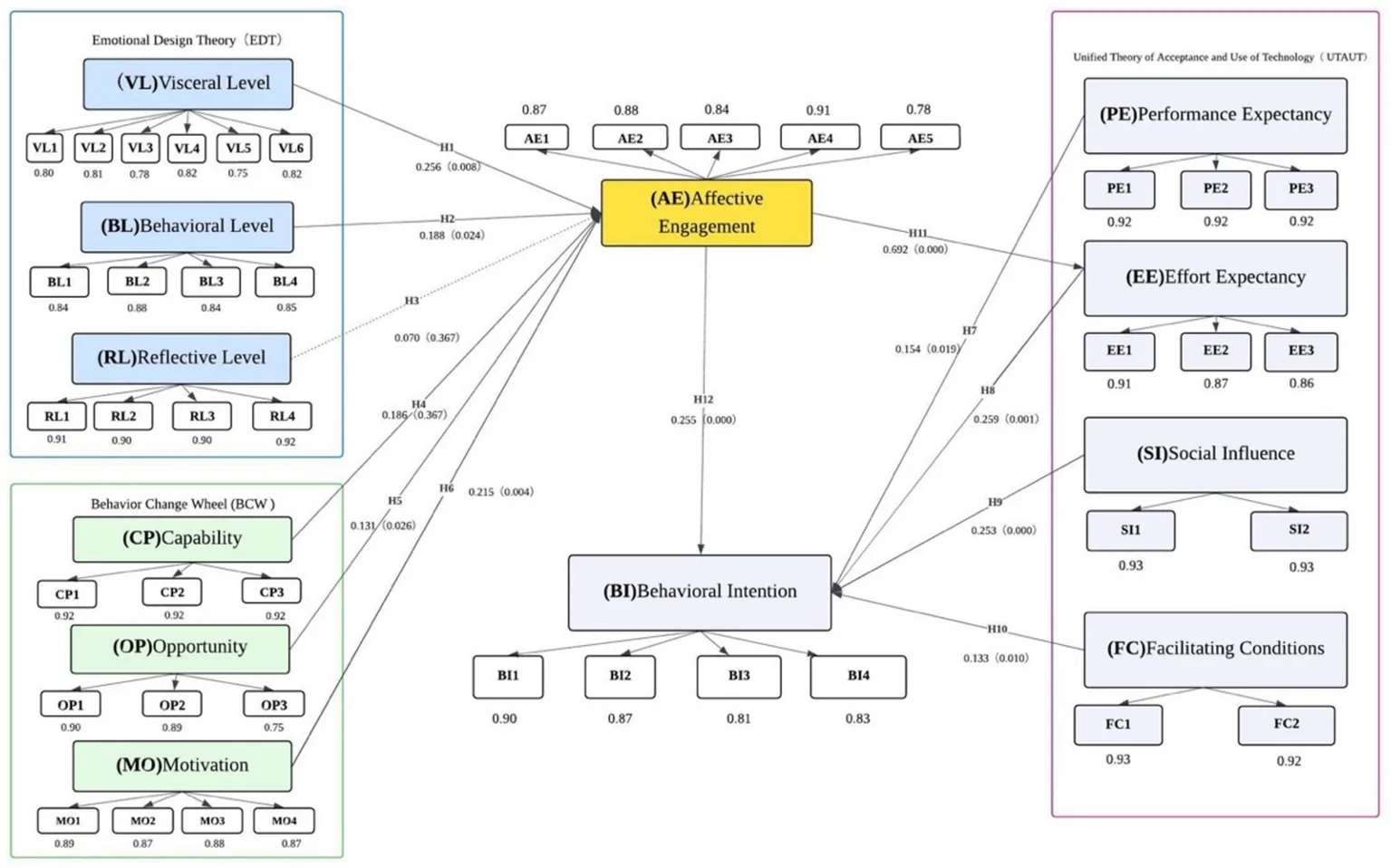
Model path analysis results.

To assess the structural model, a bias-corrected bootstrapping procedure with 5,000 resamples was employed to estimate path coefficients and their significance levels. Prior to hypothesis testing, collinearity diagnostics were conducted. All variance inflation factor (VIF) values ranged from 1.000 to 2.896, remaining below the recommended threshold of 3.3, indicating no multicollinearity concerns (Hair et al., 2019; Kock, 2015). The structural model results are presented in Table 9 and Figure 4. Among the 12 proposed hypotheses, 11 were supported. Specifically, visceral-level design (*β* = 0.256, *p* < 0.05) and behavioral-level design (β = 0.188, *p* < 0.05) significantly enhanced affective engagement, supporting H1 and H2. However, reflective-level design did not have a significant effect on affective engagement (*β* = 0.070, *p* = 0.365), resulting in the rejection of H3.

**Table 9:** Structural model results.

Hypotheses	Original sample (β)	Standard deviation	*T*	*P*	Support	VIF	
AE - > BI	0.255	0.066	3.852	***	***	2.556	0.102
AE - > EE	0.692	0.041	16.736	***	***	1.000	0.918
BL - > AE	0.188	0.083	2.254	*	Yes	2.226	0.060
CP - > AE	0.187	0.047	4.008	***	Yes	1.884	0.070
EE - > BI	0.259	0.078	3.304	**	Yes	2.237	0.120
FC - > BI	0.133	0.051	2.594	*	Yes	1.559	0.045
MO - > AE	0.215	0.075	2.877	*	Yes	2.896	0.060
OP - > AE	0.130	0.059	2.215	*	Yes	1.771	0.036
PE - > BI	0.153	0.066	2.334	*	Yes	2.596	0.036
RL - > AE	0.070	0.077	0.906	0.365	NO	--	--
SI - > BI	0.252	0.050	5.010	***	Yes	1.956	0.130
VL - > AE	0.256	0.097	2.639	*	Yes	2.720	0.090

The structural model results indicate that most hypothesized relationships were supported. Within the Emotional Design Theory (EDT) framework, visceral-level design (VL → AE; H1) and behavioral-level design (BL → AE; H2) were significantly associated with affective engagement, whereas reflective-level design (RL → AE; H3) was not significant. Under the Behavior Change Wheel (BCW), capability (CP → AE; H4), opportunity (OP → AE; H5), and emotional arousal (MO → AE; H6) were positively associated with affective engagement.

Consistent with the UTAUT framework, performance expectancy (PE → BI; H7), effort expectancy (EE → BI; H8), social influence (SI → BI; H9), and facilitating conditions (FC → BI; H10) were all significantly associated with behavioral intention. In addition, affective engagement was strongly and positively associated with effort expectancy (AE → EE; H11) and behavioral intention (AE → BI; H12).

Overall, 11 of the 12 proposed hypotheses were supported, with only H3 not supported. A detailed summary of the hypothesis testing results is provided in Table 10.

**Table 10:** Summary of hypothesis testing results.

Hypothesis	Path	*β*	*t*-value	*p*-value	Decision
H1	VL → AE	0.256	2.639	< 0.05	Supported
H2	BL → AE	0.188	2.254	< 0.05	Supported
H3	RL → AE	0.070	0.906	0.365	Not Supported
H4	CP → AE	0.187	4.008	< 0.001	Supported
H5	OP → AE	0.130	2.215	< 0.05	Supported
H6	MO → AE	0.215	2.877	< 0.05	Supported
H7	PE → BI	0.153	2.334	< 0.05	Supported
H8	EE → BI	0.259	3.304	< 0.01	Supported
H9	SI → BI	0.252	5.010	< 0.001	Supported
H10	FC → BI	0.133	2.594	< 0.05	Supported
H11	AE → EE	0.692	16.736	< 0.001	Supported
H12	AE → BI	0.255	3.852	< 0.001	Supported

### 4.3 Artificial neural network analysis

To complement the linear relationships identified through PLS-SEM, an artificial neural network (ANN) analysis was conducted to capture potential nonlinear relationships among the constructs. Following previous PLS-SEM–ANN studies, a multilayer perceptron (MLP) architecture with one hidden layer was employed. The significant predictors identified in the SEM analysis were used as input neurons, whereas the corresponding endogenous construct served as the output neuron.

**Figure 5:**
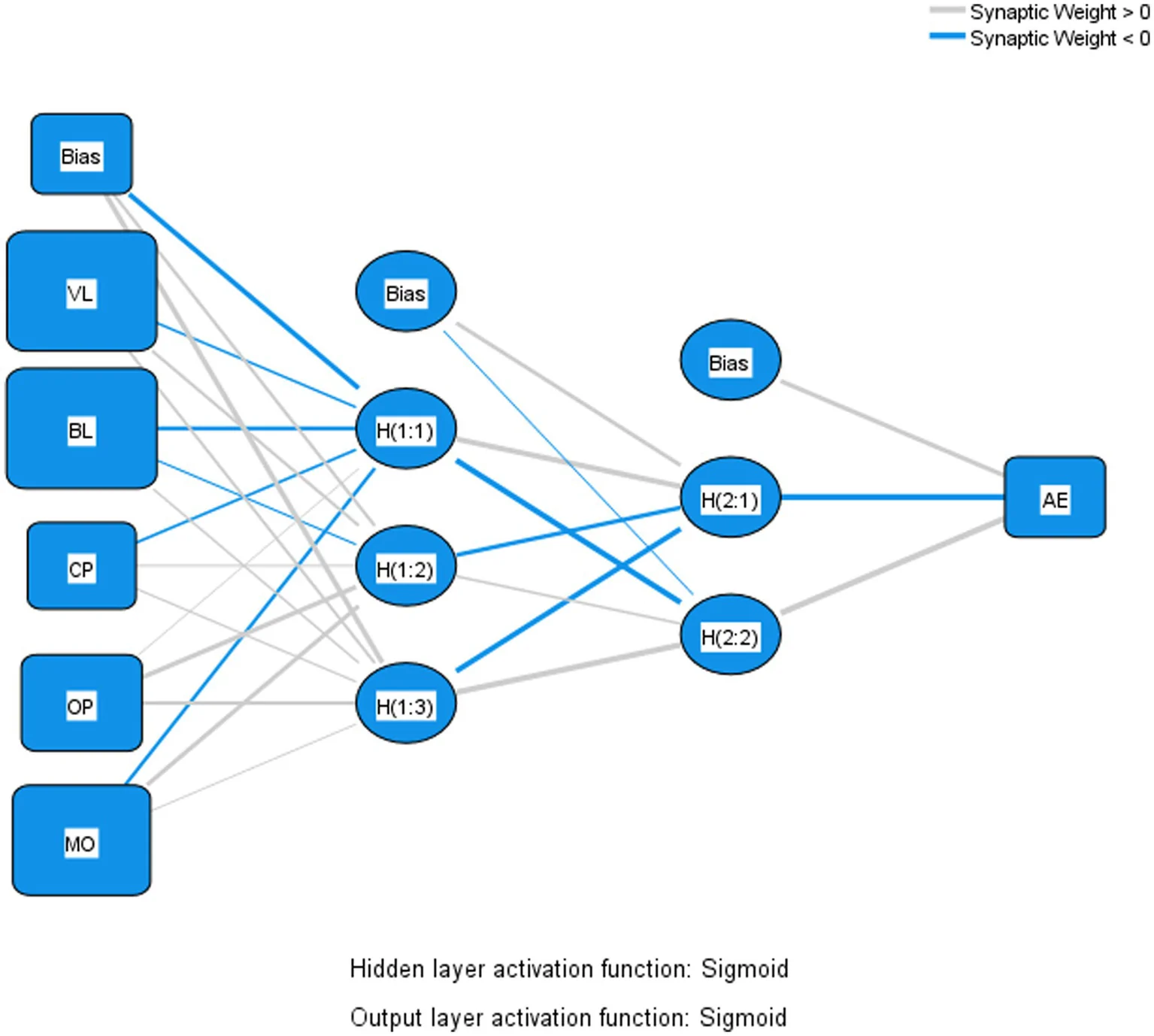
ANN Model 1 architecture (AE as output). Input variables: VL, BL, CP, OP, MO; Output variable: AE. Hidden layers use sigmoid activation functions.

The hidden layer adopted a sigmoid activation function. To evaluate predictive performance and reduce the risk of overfitting, 10 neural networks were generated using randomly partitioned training and testing datasets. Approximately 90% of the observations were used for training and the remaining 10% for testing in each run. Predictive accuracy was assessed using the root mean square error (RMSE), and the average results across the 10 runs were reported. Sensitivity analysis was subsequently performed to estimate the relative importance of the predictor variables (Tables 11, 12 and Figure 5).

**Table 11:** Model1 RMSE values.

Neural network	Training			Testing		
	Samples	SSE	RMSE	samples	SSE	RMSE
ANN1	235	1.139	0.0696	31	0.380	0.1107
ANN2	239	1.352	0.0752	27	0.136	0.0710
ANN3	242	1.415	0.0765	24	0.116	0.0695
ANN4	248	1.823	0.0857	18	0.079	0.0662
ANN5	232	1.582	0.0826	34	0.126	0.0609
ANN6	244	2.027	0.0911	22	0.092	0.0647
ANN7	239	1.294	0.0736	27	0.131	0.0697
ANN8	239	1.568	0.0810	27	0.043	0.0399
ANN9	240	1.494	0.0789	26	0.134	0.0718
ANN10	245	1.552	0.0796	21	0.139	0.0814
MEAN		1.5246	0.0794		0.1376	0.0706
SD		0.2565	0.0062		0.0906	0.0177

**Table 12:** Model1 neural network sensitivity analysis.

Neural network	VL	BL	CP	OP	MO
ANN1	0.255	0.238	0.171	0.157	0.180
ANN2	0.256	0.233	0.148	0.142	0.221
ANN3	0.281	0.266	0.152	0.122	0.179
ANN4	0.281	0.225	0.156	0.145	0.192
ANN5	0.248	0.245	0.170	0.126	0.211
ANN6	0.271	0.251	0.156	0.124	0.199
ANN7	0.247	0.207	0.184	0.156	0.206
ANN8	0.258	0.236	0.162	0.147	0.198
ANN9	0.264	0.212	0.170	0.168	0.187
ANN10	0.331	0.230	0.164	0.081	0.194
Average RI	0.269	0.234	0.163	0.137	0.197
Normalised RI (%)	100.0%	87.6%	61.1%	51.7%	73.7%
Ranking	1	2	4	5	3

RI = Relative importance.

**Figure 6:**
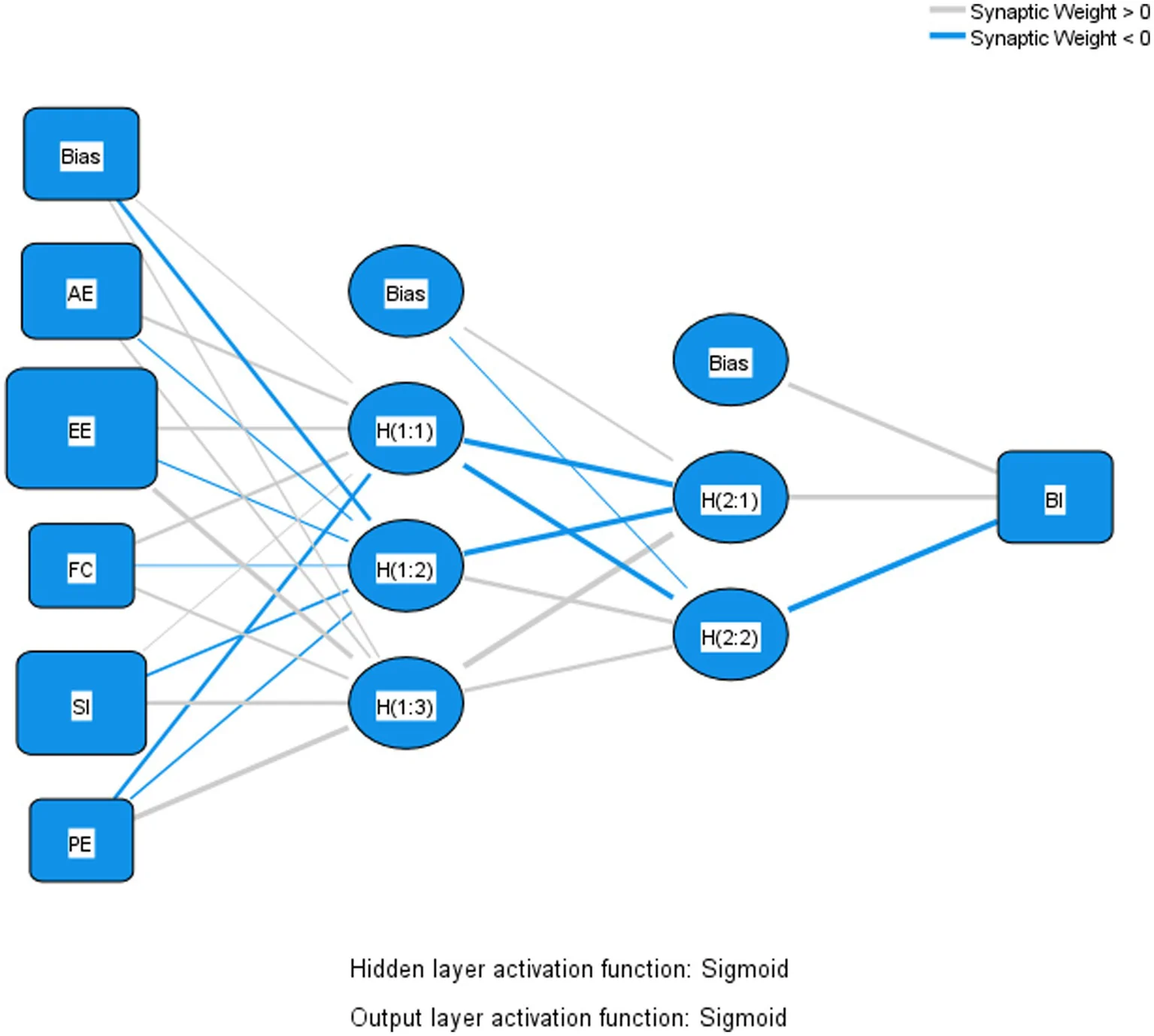
ANN Model 2 architecture (BI as output). Input variables: AE, EE, FC, SI, PE; Output variable: BI. Hidden layers use sigmoid activation functions.

For Model 2, the input variables included AE, EE, FC, SI, and PE, whereas the output variable was BI (see Figure 6). Similar to Model 1, the RMSE values for both the training and testing sets were low (Table 13), thereby indicating strong predictive accuracy. The results of the sensitivity analysis (Table 14) showed that EE exhibited the highest importance in predicting BI (100.0%), followed by AE (82.2%) and SI (67.3%), whereas PE (40.7%) and FC (31.4%) exerted comparatively weaker effects. This ranking was consistent with the ordering of path coefficients derived from the SEM, thus confirming the robustness of the structural model.

**Table 13:** Model2 RMSE values.

Neural network	Training			Testing		
	samples	SSE	RMSE	samples	SSE	RMSE
ANN1	239	1.102	0.0679	27	0.271	0.1002
ANN2	232	1.300	0.0749	34	0.177	0.0722
ANN3	242	1.752	0.0851	24	0.073	0.0552
ANN4	235	1.125	0.0692	31	0.105	0.0582
ANN5	245	1.417	0.0761	21	0.102	0.0697
ANN6	238	1.662	0.0836	28	0.417	0.0820
ANN7	241	1.133	0.0686	25	0.141	0.0751
ANN8	236	1.089	0.0679	30	0.197	0.0810
ANN9	238	1.592	0.0818	28	0.112	0.0632
ANN10	236	1.210	0.0716	30	0.081	0.0520
MEAN		1.3382	0.0747		0.1676	0.0709
SD		0.2517	0.0067		0.1066	0.0146

**Table 14:** Model 2 neural network sensitivity analysis.

Neural network	AE	EE	FC	SI	PE
ANN1	0.239	0.299	0.143	0.161	0.158
ANN2	0.267	0.290	0.064	0.244	0.135
ANN3	0.272	0.322	0.098	0.201	0.107
ANN4	0.274	0.312	0.077	0.206	0.131
ANN5	0.251	0.300	0.074	0.246	0.128
ANN6	0.246	0.348	0.017	0.243	0.147
ANN7	0.206	0.266	0.175	0.200	0.154
ANN8	0.216	0.342	0.118	0.211	0.113
ANN9	0.302	0.337	0.118	0.124	0.120
ANN10	0.291	0.313	0.076	0.255	0.065
Average RI	0.256	0.313	0.096	0.209	0.126
Normalised RI (%)	82.2%	100.0%	31.4%	67.3%	40.7%
Ranking	2	1	5	3	4

RI = Relative importance.

Overall, the ANN results revealed a variable importance ranking that was broadly consistent with the significant relationships identified by the SEM analysis. Rather than confirming causal relationships, the ANN findings provide complementary evidence regarding the relative predictive importance of the constructs and support the robustness of the observed patterns from a nonlinear prediction perspective.

### 4.4 Measurement invariance and multi-group analysis

#### 4.4.1 Measurement invariance assessment

Before conducting multi-group comparisons, the Measurement Invariance of Composite Models (MICOM) procedure was performed to assess the comparability of the measurement model across the three persona conditions. Following the guidelines proposed by Henseler et al. (2016), compositional invariance was examined using a permutation procedure. The results indicated that all constructs satisfied the requirements for compositional invariance. The original correlations between composite scores and their permuted counterparts ranged from 0.995 to 1.000 across all constructs, and all permutation *p*-values exceeded the recommended threshold of 0.05. These findings support partial measurement invariance among the Listener (Group A), Guide (Group B), and Motivator (Group C) conditions, thereby permitting meaningful comparisons of structural path coefficients across groups (Table 15).

**Table 15:** MICOM results across persona conditions.

(A VS B)	Original correlation	Correlation permutation mean	5.0%	Permutation p value
AE	1.000	1.000	0.999	0.464
BI	1.000	0.999	0.998	0.784
BL	1.000	0.999	0.997	0.912
CP	1.000	1.000	0.999	0.747
EE	1.000	0.999	0.998	0.423
FC	1.000	0.999	0.997	0.694
MO	1.000	1.000	0.999	0.808
OP	0.998	0.997	0.990	0.418
PE	1.000	1.000	0.999	0.601
RL	1.000	1.000	1.000	0.346
SI	0.999	0.999	0.998	0.090
VL	0.999	0.999	0.997	0.611

(A VS C)	Original correlation	Correlation permutation mean	5.0%	Permutation *p-*value
AE	1.000	1.000	0.999	0.540
BI	1.000	1.000	0.999	0.192
BL	1.000	0.999	0.997	0.969
CP	1.000	1.000	0.999	0.234
EE	0.999	0.999	0.998	0.209
FC	1.000	0.999	0.997	0.751
MO	1.000	0.999	0.998	0.718
OP	1.000	0.999	0.997	0.683
PE	1.000	1.000	0.999	0.483
RL	1.000	1.000	0.999	0.104
SI	1.000	1.000	0.999	0.674
VL	0.998	0.999	0.997	0.200

(B VS C)	Original correlation	Correlation permutation mean	5.0%	Permutation *p-*value
AE	1.000	1.000	0.998	0.651
BI	1.000	0.999	0.999	0.507
BL	1.000	0.999	0.998	0.962
CP	1.000	1.000	0.999	0.684
EE	1.000	0.999	0.998	0.752
FC	1.000	1.000	0.998	0.977
MO	1.000	0.999	0.998	0.894
OP	0.995	0.998	0.993	0.117
PE	1.000	1.000	0.999	0.893
RL	1.000	1.000	1.000	0.818
SI	0.999	0.999	0.998	0.248
VL	0.999	0.999	0.998	0.645

Overall, the MICOM results suggest that the measurement model operated equivalently across persona conditions and that subsequent multi-group analysis could be conducted with confidence.

#### 4.4.2 PLS-MGA results

To investigate whether the proposed acceptance mechanism differed across persona styles, a Partial Least Squares Multi-Group Analysis (PLS-MGA) was conducted. Pairwise comparisons were performed among the Listener (A), Guide (B), and Motivator (C) groups. The results are presented in Tables 16–18.

**Table 16:** PLS-MGA results: Listener (A) versus guide (B).

Relationship	Difference (Group_A - Group_B)	2-tailed significance (*p*-value)
AE - > BI	−0.353	0.024
AE - > EE	−0.136	0.195
BL - > AE	−0.209	0.315
CP - > AE	0.148	0.241
EE - > BI	0.210	0.279
FC - > BI	−0.099	0.396
MO - > AE	−0.205	0.257
OP - > AE	0.150	0.382
PE - > BI	0.133	0.396
RL - > AE	−0.154	0.480
SI - > BI	0.106	0.392
VL - > AE	0.243	0.210
MO - > AE - > BI	−0.142	0.018

**Table 17:** PLS-MGA results: Listener (A) versus motivator (C).

Relationship	Difference (Group_B - Group_C)	2-tailed significance (*p*-value)
AE - > BI	−0.414	0.006
AE - > EE	−0.144	0.139
BL - > AE	−0.281	0.191
CP - > AE	0.122	0.413
EE - > BI	0.037	0.834
FC - > BI	0.125	0.292
MO - > AE	0.074	0.657
OP - > AE	−0.142	0.347
PE - > BI	0.259	0.101
RL - > AE	0.030	0.851
SI - > BI	0.033	0.790
VL - > AE	0.090	0.684
MO - > AE - > BI	−0.034	0.594

**Table 18:** PLS-MGA results: Guide (B) versus motivator (C).

Relationship	Difference (Group_A - Group_C)	2-tailed significance (*p*-value)
AE - > BI	−0.061	0.720
AE - > EE	−0.008	0.943
BL - > AE	−0.072	0.786
CP - > AE	−0.026	0.818
EE - > BI	−0.173	0.332
FC - > BI	0.224	0.091
MO - > AE	0.279	0.086
OP - > AE	−0.292	0.062
PE - > BI	0.126	0.392
RL - > AE	0.184	0.325
SI - > BI	−0.073	0.506
VL - > AE	−0.153	0.469
MO - > AE - > BI	0.108	0.193

##### 4.4.2.1 Listener (A) versus guide (B)

The comparison between the Listener and Guide conditions revealed several significant differences. Specifically, the relationship between affective engagement and behavioral intention was significantly stronger in the Guide condition than in the Listener condition (Δβ = −0.353, *p* = 0.024). In addition, the indirect effect of emotional arousal on behavioral intention through affective engagement was significantly stronger in the Guide condition (Δβ = −0.142, *p* = 0.018). No significant differences were observed for the remaining structural paths.

##### 4.4.2.2 Listener (A) versus motivator (C)

The comparison between the Listener and Motivator conditions identified a significant difference in the path from affective engagement to behavioral intention (Δβ = −0.414, *p* = 0.006). The effect was stronger in the Motivator condition, indicating that emotionally engaged users were more likely to develop behavioral intentions when interacting with a motivational persona. No other significant differences were detected.

##### 4.4.2.3 Guide (B) versus motivator (C)

No statistically significant differences were found between the Guide and Motivator conditions for any of the examined structural relationships. This finding suggests that the two persona styles produced highly similar acceptance mechanisms despite differences in communication style.

Overall, the MGA results indicate that the proposed acceptance mechanism remained largely stable across persona styles, as most structural relationships did not differ significantly among groups. However, significant differences emerged in the strength of the relationship between affective engagement and behavioral intention. Compared with the Listener persona, both the Guide and Motivator personas demonstrated a stronger translation of affective engagement into behavioral intention. These findings suggest that persona styles do not fundamentally alter the overall acceptance mechanism but may influence the magnitude of key psychological relationships within the acceptance process.

## 5 Discussion

### 5.1 Discussion of significant paths

The findings indicate that, in the emotionally sensitive context of mental health interventions, university students’ behavioral intention toward AI-based mental health chatbots is not driven by a single mechanism. Instead, it appears to emerge from the joint influence of an affect-driven pathway and a cognition-based evaluation pathway. This pattern suggests that, when users are under emotional vulnerability, their responses may be shaped by a more complex interplay of affective and cognitive processes rather than by cognition alone.

Looking more closely at the affect-driven pathway, both visceral-level and behavioral-level design features were associated with higher levels of affective engagement, which was in turn associated with greater behavioral intention. Prior studies have shown that visual style and anthropomorphic appearance can trigger immediate emotional reactions through rapid affective appraisal (Norman, 2013). At the same time, elements such as linguistic tone and interaction rhythm tend to shape users’ ongoing experience by improving cognitive fluency and interaction coherence (Nass and Moon, 2000; Niculescu et al., 2013).

In the present study, these mechanisms do not seem to operate in isolation. Rather, they appear to unfold as a continuous process, where initial emotional activation gradually develops into affective resonance. Together, these processes shape how users perceive and engage with the system. This differs from more traditional information system settings, where interface design is often treated as secondary. Here, design cues seem to play a much more central role in driving user acceptance.

Contrary to expectations, reflective-level design was not significantly associated with affective engagement. According to Emotional Design Theory, reflective processing represents a higher-order evaluative process involving meaning-making, self-reflection, and the interpretation of interaction experiences. In the present study, participants were exposed to a single scenario-based interaction rather than engaging in sustained or repeated use of the chatbot. Under such conditions, users may have relied primarily on immediate perceptual impressions and interaction experiences when forming affective responses, while deeper reflective evaluations had limited opportunity to develop. This finding suggests that affective engagement in early-stage mental health chatbot interactions may be driven more strongly by visceral and behavioral design cues than by reflective-level considerations. Rather than indicating that reflective processes are unimportant, the result implies that their influence may emerge more clearly during longer-term interactions and continued use of AI-based mental health systems.

Turning to the cognition-based evaluation pathway, capability, opportunity, and motivation were not found to directly predict acceptance. Instead, their effects appear to operate through affective engagement. Earlier work has suggested that these factors function as basic conditions for behavioral participation (Fancourt et al., 2020). However, the current findings imply that, in human–AI interaction contexts, these conditions may first influence users’ emotional states and sense of involvement before shaping acceptance outcomes. In other words, enabling conditions do not translate directly into behavior but seem to work through users’ experiential responses.

A similar pattern can be observed for UTAUT-related constructs. Effort expectancy and social influence show relatively strong associations with behavioral intention, while performance expectancy and facilitating conditions play a less prominent role. This differs from traditional technology acceptance research, where performance expectancy is often considered the dominant predictor (Venkatesh et al., 2003). One possible explanation is that users in this context do not primarily approach chatbots as problem-solving tools. Instead, they appear to engage with them as sources of emotional support. The effect of social influence further suggests that perceived social support and reduced stigma may play an important role in shaping acceptance in mental health settings (Yamaguchi et al., 2019).

Affective engagement emerges as a key mechanism across the model. Affective engagement was positively associated with effort expectancy, while both affective engagement and effort expectancy were associated with behavioral intention. This suggests that even if a system is not entirely effortless to use, users may still be willing to engage when they feel emotionally connected. This observation is broadly consistent with earlier findings indicating that emotional expressiveness can offset usability constraints to some extent (Bickmore et al., 2005). On the other hand, mediation results indicate that emotional arousal influences behavioral intention through affective engagement, forming a process that can be understood as “emotional activation → affective experience → acceptance.” This aligns with emotion regulation perspectives, which suggest that people rely more on affective processing under stress.

From a methodological standpoint, the ANN results revealed a variable importance ranking that was broadly consistent with the significant relationships identified by the SEM analysis. Rather than confirming causal relationships, the ANN findings provide complementary nonlinear predictive evidence regarding the relative importance of the constructs and support the robustness of the observed patterns from a predictive perspective.

Overall, the findings point to a shift in how user acceptance should be understood in mental health chatbot contexts. Rather than being primarily driven by perceived usefulness, behavioral intention appears to be closely tied to users’ affective experiences. In this sense, feeling understood may matter as much as, or even more than, whether the system is perceived as useful. Nevertheless, this interpretation should be considered within the context of a university student sample. The relative importance of affective experiences compared with cognitive evaluations may vary across age groups, psychological conditions, and cultural settings.

While the preceding discussion focuses on the overall acceptance mechanism estimated across the full sample, the experimental design of this study also allows for the examination of potential differences across persona styles. To address RQ1 and evaluate whether the proposed relationships vary according to the Listener, Guide, and Motivator conditions, an additional multi-group analysis was conducted. The results provide further insight into how persona styles influence the strength of key psychological relationships within the acceptance process and are discussed in the following section.

### 5.2 Discussion of persona style differences

Beyond the overall structural relationships, the multi-group analysis provides additional insights into how different persona styles shape the acceptance process. Although the overall acceptance mechanism remained largely stable across the Listener, Guide, and Motivator conditions, several significant differences emerged in the strength of specific relationships.

Most notably, the relationship between affective engagement and behavioral intention was significantly stronger in both the Guide and Motivator conditions than in the Listener condition. This finding suggests that while affective engagement is important across all persona styles, its association with behavioral intention varies according to the interaction style adopted by the virtual agent. Users interacting with Guide and Motivator personas appeared more likely to translate positive affective experiences into intentions to continue using the chatbot.

One possible explanation is that the Guide persona provides users with a clearer sense of structure and problem-solving support. In mental health contexts, emotional support alone may not always be sufficient to encourage continued engagement. Users may also seek practical guidance and actionable suggestions. By combining emotional responsiveness with structured support, the Guide persona may strengthen the association between affective engagement and behavioral intention.

A similar explanation may account for the Motivator persona. Unlike the Listener, which primarily focuses on emotional validation, the Motivator actively encourages users to take positive actions and maintain confidence. Previous studies on persuasive technologies suggest that positive reinforcement can enhance users’ perceived self-efficacy and willingness to act. Consequently, affective engagement generated by the Motivator persona may be more readily associated with future usage intentions.

Interestingly, no significant differences were observed between the Guide and Motivator conditions. This finding suggests that although the two personas employ different communication styles, they may operate through similar psychological mechanisms. Both personas extend beyond passive emotional support and provide users with a sense of behavioral direction, either through structured guidance or motivational encouragement.

Overall, the MGA results suggest that persona styles do not fundamentally alter the proposed acceptance mechanism. Rather, persona styles appear to influence the strength of key relationships within the mechanism. Therefore, persona design may be understood as a contextual factor that shapes how affective experiences are translated into behavioral intentions under experimental exposure conditions. Therefore, the observed differences should be interpreted as variations in the strength of associations under experimental exposure conditions rather than evidence that one persona style is universally superior to another.

However, these findings should be interpreted within the context of the present sample, which consisted primarily of young university students. The relative effectiveness of Listener, Guide, and Motivator personas may differ among older adults, clinical populations, or individuals experiencing severe psychological distress, whose support needs and interaction preferences may not be comparable to those of university students. Consequently, the observed persona-related differences should not be assumed to generalize across all mental health user groups.

### 5.3 Discussion of non-significant paths

The results show that H3 (RL → AE, *β* = 0.070, *t* = 0.906, *p* = 0.365) was not statistically significant. In other words, reflective-level perceptions of cross-modal consistency did not appear to have a meaningful effect on affective engagement in this context. At first glance, this seems to contrast with prior studies suggesting that consistency across visual, linguistic, and behavioral cues enhances trust, immersion, and perceived system credibility (Kim and Sundar, 2012; Nass and Moon, 2000; Niculescu et al., 2013; Yamaguchi et al., 2019). However, a closer look suggests that this difference may be related to how users process information under different interaction conditions.

Much of the earlier work on cross-modal consistency has been conducted in task-oriented or information-processing settings. In those contexts, users tend to evaluate systems more deliberately, and consistency often serves as a cue for reliability and coherence. Under such conditions, maintaining alignment across modalities becomes an important basis for trust formation. By contrast, the present study focuses on a mental health intervention scenario, where emotional salience is relatively high and cognitive resources may be more limited.

From this perspective, users in emotionally demanding situations may rely less on reflective evaluation and more on immediate, affective responses. Dual-process theories suggest that when individuals are under stress, they are more likely to depend on heuristic and emotion-based processing rather than systematic reasoning (Kahneman, 2011; Petty and Cacioppo, 1986). In such cases, higher-order design features—such as cross-modal consistency—may receive less attention compared with more immediate cues related to emotional tone and responsiveness.

This explanation also fits with the functional purpose of interaction in mental health contexts. Here, users are not primarily trying to complete tasks or verify information accuracy. Instead, they are often seeking emotional relief and reassurance. As a result, system evaluation may be based more on whether the interaction feels supportive and responsive than on whether different modalities are fully aligned. This shift in focus moves attention away from reflective-level processing toward visceral and behavioral experiences, which could help explain why consistency does not show a strong effect on engagement.

User characteristics may further reinforce this pattern. The present findings may reflect the characteristics of university students, who often engage with digital technologies in ways that emphasize immediacy and responsiveness. At the same time, their reliance on systematic evaluation strategies may be relatively lower. It is also possible that participants focused more on immediate interaction cues than on higher-order consistency cues during the brief experimental exposure. It is also possible that aspects of the experimental design influenced the result. In this study, visual and linguistic features were kept relatively consistent across conditions in order to avoid confounding effects. While this improves internal validity, it may also reduce the observable variation in perceived inconsistency, thereby weakening its statistical effect. Future research could address this by introducing more explicit manipulations of cross-modal consistency.

Taken together, the non-significant result for H3 should not be taken to mean that consistency is irrelevant. Rather, it suggests that its influence may depend on the interaction context, users’ cognitive state, and the goals of the interaction. In emotionally driven settings such as mental health support, users may place greater weight on immediate emotional responsiveness than on reflective-level consistency, which in turn reshapes the relative importance of different design features.

### 5.4 Design implications

The findings of this study indicate that, in mental health contexts where users are emotionally vulnerable, acceptance of virtual agents is shaped more by affective experience than by purely cognitive evaluation. This pattern suggests that design decisions should not focus only on improving system functionality. Instead, designers need to pay closer attention to how interaction experiences are felt and interpreted by users during use.

One implication concerns the role of emotional immediacy in early-stage interaction. The results show that cues at the visceral and behavioral levels influence how users initially engage with the system. Visual appearance and interaction style, for example, can shape users’ emotional reactions within a very short time frame. When users first encounter a chatbot, they are unlikely to evaluate all its functions in detail; instead, they respond to whether the interaction feels approachable and responsive. For this reason, it may be more effective to reduce emotional barriers through simple visual styles and empathetic language, rather than attempting to present a wide range of features at the outset.

Another implication relates to cross-modal consistency. Previous studies often treat consistency as a general requirement for interface design, but the present findings suggest that its importance may depend on how and when the system is used. In short-term interactions that focus on emotional support, users may pay less attention to whether visual, linguistic, and behavioral elements are fully aligned. What matters more in these situations is whether the system responds in a way that feels timely and supportive. Over longer periods of use, however, consistency may become more relevant, as users begin to form more stable expectations about the system. This suggests that designers should consider different time horizons when deciding how much emphasis to place on consistency.

Persona design may benefit from flexibility, as different agent roles appear to be associated with different user responses depending on users’ needs and situational contexts. Rather than treating persona as fixed, future systems could adapt interaction styles based on user state and context. At the same time, these design implications should be interpreted cautiously. Because the present study focused exclusively on university students, the identified design preferences may reflect the characteristics of younger and digitally experienced users. Different populations, such as older adults, clinical patients, or users from different cultural backgrounds, may respond differently to persona-based interaction strategies. Therefore, future design guidelines should be validated across broader user groups before being generalized to mental health chatbot applications more broadly.

The role of theory should also be interpreted cautiously. Frameworks such as Emotional Design Theory and the Behavior Change Wheel help explain user responses but do not directly prescribe design solutions. Their value lies in informing underlying processes rather than specific features.

Overall, mental health chatbot design should move beyond feature-driven approaches and focus more on user experience, particularly whether users feel understood and able to engage.

### 5.5 Limitations and ethical considerations of AI chatbots in mental health interventions

Although AI chatbots offer advantages in accessibility, low entry barriers, and anonymity, their actual therapeutic effectiveness remains limited in practice. The present study focuses on university students’ interaction experiences related to emotional support and behavioral intention toward mental health chatbots. Therefore, the findings should not be interpreted as evidence that AI chatbots can replace professional psychotherapy or clinical intervention. Rather, they should be understood as reflecting the potential role of such systems as supplementary tools within broader mental health care settings.

It is important to interpret the findings within the scope of the present research design. This study employed a cross-sectional scenario-based experiment and relied on participants’ self-reported perceptions following exposure to standardized dialogue scenarios. Therefore, the observed relationships should be interpreted as associations under experimental exposure rather than definitive evidence of long-term causal effects. Although the findings suggest that persona styles are associated with affective engagement and behavioral intention, the present design does not permit conclusions regarding long-term acceptance, sustained usage behavior, or clinical effectiveness. Future research should employ longitudinal designs, field experiments, and real-world usage data to examine whether these relationships persist over time and translate into actual behavioral outcomes.

The sample in this study was limited to university students, which may restrict the generalizability of the findings. At the same time, the focus on this group may also influence the mechanism observed in this study. Many university students tend to prioritize immediacy, emotional responsiveness, and low interaction barriers, which may help explain why affective pathways appear more prominent in the results. In contrast, other populations, including older adults, clinical patients, individuals experiencing severe psychological distress, and users from different cultural backgrounds, may rely on different evaluation criteria and interaction preferences. Future research should therefore examine whether the relative importance of affective and cognitive pathways varies across different populations, psychological conditions, and cultural contexts.

The cross-sectional design limits the ability to capture how user responses evolve over time. Affective engagement and psychological acceptance may shift with continued interaction, and future research could use longitudinal or real-world data to better understand these changes.

Ethical concerns extend beyond data privacy to include psychological dependence. While affective engagement supports acceptance, highly engaging systems may lead to overreliance and delay professional help. Future research should examine the boundary between beneficial engagement and problematic dependence.

Although AI chatbots may reduce stigma, they may also influence users’ real-world social behavior. Emotional responsiveness should therefore be balanced with transparency, ensuring that users recognize the system’s supportive—rather than substitutive—role and remain connected to human support.

Accordingly, the findings should be interpreted as evidence derived from a university student population under experimental exposure conditions and should not be generalized to broader mental health populations without further empirical validation.

Overall, the value and risks of AI chatbots in mental health depend on context, and their role should remain supportive rather than substitutive within broader care systems.

## 6 Conclusion

### 6.1 Theoretical implications

This study contributes to the literature by integrating existing theoretical perspectives within a specific application context, rather than proposing a new causal model. The findings show that Emotional Design Theory (EDT) and the Behavior Change Wheel (BCW) can be reinterpreted as useful analytical lenses for understanding user responses in AI-based mental health interactions. Instead of functioning as standalone predictive models, these frameworks are used here to capture different but complementary aspects of the interaction process. In this study, design features operating at the visceral and behavioral levels shape users’ immediate affective reactions and ongoing engagement experiences, while the capability–opportunity–motivation structure helps explain the conditions under which users are more or less likely to engage in psychological support interactions.

Based on this perspective, the study also extends the use of the Unified Theory of Acceptance and Use of Technology (UTAUT) in emotionally sensitive contexts. In this study, behavioral intention was used as the primary outcome variable representing users’ willingness to continue using mental health chatbots. This focus allows the study to capture acceptance-related evaluations while remaining consistent with the original UTAUT framework. At the same time, motivation within the BCW framework is operationalized as emotional arousal, which reflects how immediate affective activation influences early-stage engagement. These adjustments do not change the core structure of the original models, but they make them more suitable for explaining user responses in mental health interaction settings.

One key contribution of this study is the identification of affective engagement as a central process-level mechanism. Instead of treating affective engagement simply as an outcome of design features or as a substitute for behavioral intention, it is conceptualized as an intermediate process that links together emotional activation, interaction experience, and behavioral intention. This perspective adds to existing research by shifting attention from static relationships between variables to the dynamic processes through which users form responses during interaction. It also provides a clearer understanding of how affective and cognitive processes jointly contribute to behavioral intention toward mental health chatbots.

An additional theoretical implication concerns the non-significant effect of reflective-level design on affective engagement. According to Emotional Design Theory, reflective processing represents a higher-order evaluative process involving meaning-making, self-reflection, and the interpretation of interaction experiences. In the present study, participants were exposed to a single scenario-based interaction rather than engaging in sustained or repeated use of the chatbot. Under such conditions, users may have relied primarily on immediate perceptual impressions and interaction experiences when forming affective responses, while deeper reflective evaluations had limited opportunity to develop. This finding suggests that affective engagement in early-stage mental health chatbot interactions may be driven more strongly by visceral and behavioral design cues than by reflective-level considerations. Rather than indicating that reflective processes are unimportant, the result implies that their influence may emerge more clearly during longer-term interactions, relationship development, and continued use of AI-based mental health systems.

The findings also speak to ongoing debates in technology acceptance research. They suggest that, in emotionally sensitive contexts, affective processes may play a more central role than purely cognitive evaluations. This challenges the traditional utility-centered assumptions of models such as UTAUT and points to the need for a more balanced perspective that incorporates both emotional and experiential dimensions of user interaction. It also offers a basis for future research on technology acceptance in contexts characterized by emotional vulnerability, where feeling “understood” can be just as important as perceived usefulness.

At the same time, these theoretical implications should be interpreted in relation to the characteristics of the study sample. The relative importance of affective and cognitive mechanisms observed in this study may vary across populations with different psychological needs, demographic characteristics, and cultural backgrounds. Future research is needed to examine the boundary conditions of the proposed mechanism across diverse user groups.

### 6.2 Practical implications

In practice, this study offers practical insights for the design of AI chatbots in university mental health services, with a focus on the role of affective processes in shaping user acceptance. The findings show that many students tend to prioritize emotional immediacy and interaction simplicity over functional complexity and external facilitating conditions. This pattern suggests a shift in design priorities. Rather than focusing on feature richness, chatbot systems in mental health contexts need to reduce emotional barriers and enhance users’ sense of psychological safety.

Designers need to prioritize affective accessibility as a core interaction principle. Affiliative visual cues, emotionally warm language, and feedback that is sensitive to users’ affective states can help users engage emotionally at an early stage by lowering initial resistance and creating a sense of being understood. At a more detailed level, these features operate at the visceral and behavioral levels, triggering immediate emotional responses and sustaining engagement through perceived responsiveness. In early interactions, this means that design should aim to reduce cognitive load while supporting emotional resonance, especially in low-risk and short-duration support scenarios.

Persona design can be understood as a context-dependent system rather than a fixed stylistic choice. The findings suggest that different persona styles serve different psychological functions. Listener-oriented agents tend to support emotional expression and companionship, while guide- or motivator-oriented agents are more suitable for goal-oriented activities such as stress management or learning. This points to the need to align persona configurations with users’ situational needs and psychological states. Designers could implement this through adaptive systems that adjust interaction styles based on user input, emotional signals, or usage context, instead of relying on a single static design.

The role of cross-modal consistency appears to depend on context. In short-term, affect-driven interactions, users focus more on immediacy and emotional reassurance, which reduces the importance of consistency. In contrast, consistency becomes more relevant in long-term interactions for building trust and supporting continued engagement.

AI chatbots are better positioned as supportive tools within broader mental health systems rather than standalone solutions. Their value lies in lowering help-seeking barriers, providing immediate support, and encouraging early engagement. Design should define system boundaries and include referral mechanisms to human professionals when needed.

These practical implications should be interpreted within the scope of the present study population. Because the findings were derived from university students, the identified design preferences may primarily reflect the characteristics of younger and digitally experienced users. Older adults, clinical populations, individuals experiencing severe psychological distress, and users from different cultural backgrounds may have different expectations regarding emotional support, usability, and trust. Therefore, the design recommendations proposed in this study should be adapted cautiously when applied to broader mental health contexts and should be validated through future research involving more diverse user groups.

Overall, effective chatbot design in mental health contexts should move from function-oriented development toward experience-oriented design, with emphasis on emotional engagement, contextual adaptation, and system integration.

### 6.3 Limitations and future directions

Although this study offers useful insights into user acceptance of AI-based mental health chatbots, several limitations should be acknowledged, which also suggest directions for future research.

First, the data were collected from a single university, which may limit the diversity of the sample and affect the generalizability of the findings. In addition, participation in the study was voluntary, creating the possibility of self-selection bias. Individuals who were more interested in AI technologies, mental health topics, or digital support services may have been more likely to participate than those with lower levels of interest. Consequently, the sample may not fully represent the broader university student population. The stronger role of the affect-driven pathway observed in this study may partly reflect the characteristics of university students, who tend to prioritize immediacy, emotional responsiveness, and low interaction barriers. Different user groups may exhibit different patterns of technology acceptance. For example, older adults, clinical populations, individuals experiencing severe psychological distress, or users from different cultural backgrounds may place greater emphasis on perceived usefulness, trust, or long-term effectiveness. Future studies should recruit participants from multiple institutions and employ more diverse sampling strategies to improve representativeness and external validity.

Second, the cross-sectional design makes it difficult to observe how user responses develop over time. Affective engagement and behavioral intention are not fixed and may change as users become more familiar with the system. While emotional responsiveness may shape initial impressions, factors such as consistency, reliability, and perceived effectiveness may gradually become more important. Future research using longitudinal designs, experience sampling methods, or real-world usage data could provide a more comprehensive understanding of how these processes evolve during continued interaction.

Third, all constructs were measured using self-reported responses collected through a single post-interaction questionnaire. Although the full-collinearity assessment suggested that common method bias was unlikely to represent a serious threat to the validity of the findings, method-related variance cannot be completely ruled out. Future research could strengthen methodological rigor by incorporating marker-variable techniques, behavioral indicators, multi-source measures, or longitudinal data collection procedures.

In addition, the directional relationship between affective engagement and effort expectancy should be interpreted cautiously. Although the present model specifies affective engagement as an antecedent of effort expectancy, alternative causal orderings are also plausible. Because the present study relies on cross-sectional self-report data, it cannot establish temporal precedence between these constructs. Therefore, the observed relationship should be interpreted as supporting a theoretically specified association within the proposed model rather than confirming a definitive causal direction. Future longitudinal, experimental, or cross-lagged studies are needed to determine whether affective engagement precedes effort expectancy, whether effort expectancy facilitates affective engagement, or whether the relationship is reciprocal.

Fourth, the use of a semi-anthropomorphic virtual agent limits the examination of other forms of anthropomorphism. Future research could manipulate additional design features, such as voice characteristics, facial expressions, embodiment, and multimodal interaction cues, to better understand their influence on user experience and acceptance in mental health contexts.

Fifth, although constructs from UTAUT and the BCW were adapted to fit the mental health chatbot context, some dimensions—particularly social influence and facilitating conditions—may not fully capture the affective and social dynamics involved in human–AI interactions. Future studies may benefit from incorporating alternative theoretical frameworks that place greater emphasis on emotional, relational, and experiential processes.

Finally, emerging digital health technologies, including wearable devices, multimodal sensing systems, and adaptive AI services, offer new opportunities for continuous and personalized mental health support. Future research could explore how mental health chatbots can be integrated with these technologies to provide more context-aware, personalized, and sustainable intervention experiences.

## Data Availability

The original contributions presented in the study are included in the article/Supplementary material, further inquiries can be directed to the corresponding author/s.
