# Supplementary material for: When chatbots are perceived as “understanding”: a study on how virtual agent persona styles and affective interaction promote mental health acceptance
Source: Front Psychol. 2026 Sep 3;17:1863595. doi: 10.3389/fpsyg.2026.1863595 (PMC13582545; doi:10.3389/fpsyg.2026.1863595)
Supplement: Supplementary file 1 [file Data_Sheet_1.docx]

# Supplementary File S1

## Complete Experimental Dialogue Scripts for the Three Persona Conditions

Note. The dialogue excerpts shown below are representative examples of the standardized persona stimuli used in the experiment. Across all conditions, the interaction topic, dialogue structure, and conversational length were kept consistent. Differences were limited to persona-specific communication styles and feedback strategies.

**Appendix A. Listener Persona Condition**

Persona Characteristics:

• Deep Listening

• Empathic Companionship

• Emotional Support

**Round 1:**

Listener: Hello, nice to meet you.

User: Hello.

**Round 2:**

Listener: How are you feeling today?

User: A little tired.

**Round 3:**

Listener: Would you like to talk to me?

User: Sure.

**Round 4:**

Listener: I will listen carefully.

User: Hearing you say that makes me feel at ease.

**Round 5:**

Listener: Shall we talk slowly?

User: Okay, I'm willing to explain slowly.

**Round 6:**

Listener: Thank you for sharing these feelings with me. Whenever you need to talk, I will patiently accompany you.

User: Thank you, I feel a little more relaxed.

**Appendix B. Guide Persona Condition**

Persona Characteristics:

• Structured Guidance

• Problem Analysis

• Goal Orientation

**Round 1:**

Guide: Hello. I am your emotional navigator.

User: Hello.

**Round 2:**

Guide: Have you encountered any difficulties lately?

User: Some things are making me anxious.

**Round 3:**

Guide: Let's break it down step by step.

User: Okay.

**Round 4:**

Guide: Tell me what's worrying you.

User: The pressure of studying is too much.

**Round 5:**

Guide: Let's find solutions together.

User: Okay, I want to know how to do it.

**Round 6:**

Guide: Today we've identified the main problems and possible solutions. Now you can try them step by step, and I believe you can gradually improve your current situation.

User: Okay, I'll try to follow these methods.

**Appendix C. Motivator Persona Condition**

Persona Characteristics:

• Positive Reinforcement

• Encouragement

• Action Orientation

**Round 1:**

Motivator: Hi! You look great, want to chat for a bit?

User: Really?

**Round 2:**

Motivator: Yes, you're very powerful.

User: But I'm a little worried

**Round 3:**

Motivator: Challenges are just opportunities for growth.

User: You're right.

**Round 4:**

Motivator: I'll always be cheering you on!

User: Thank you for your encouragement!

**Round 5:**

Motivator: Let's overcome difficulties together!

User: Okay, I will try to face it.

**Round 6:**

Motivator: You've taken a positive step, and I believe you have the ability to face challenges. Keep your confidence up, I'll be cheering you on!

User: Thank you, I'll keep trying!
